# Prediction of higher ceftazidime–avibactam concentrations in the human renal interstitium compared with unbound plasma using a minimal physiologically based pharmacokinetic model developed in rats and pigs through microdialysis

**DOI:** 10.1128/aac.01518-24

**Published:** 2025-02-06

**Authors:** Maxime Vallée, Vincent Aranzana-Climent, Jérémy Moreau, Isabelle Lamarche, Théo Fontanier, Céline Barc, Nathalie Kasal-Hoc, Céline Debiais-Delpech, Hélène Mirfendereski, Jérémy Pezant, Anne Pinard, Jonathan Clarhaut, William Couet, France Cazenave-Roblot, Sandrine Marchand

**Affiliations:** 1 Université de Poitiers, INSERM U1070, PHAR2 27077 , Poitiers, France; 2 Département d’urologie et de transplantation rénale, Centre Hospitalier Universitaire 36655 , Poitiers, France; 3 Plateforme d’Infectiologie Expérimentale (PFIE), UE1277, INRAE Centre Val de Loire 56586 , Nouzilly, France; 4 Service d’anatomopathologie, CHU de Poitiers 36655 , Poitiers, France; 5 Laboratoire de Toxicologie et de Pharmacocinétique, CHU de Poitiers 36655 , Poitiers, France; 6 Service de Maladies Infectieuses et Tropicales, CHU de Poitiers 36655 , Poitiers, France; Providence Portland Medical Center, Portland, Oregon, USA

**Keywords:** ceftazidime–avibactam, urinary tract infection, pharmacokinetics, microdialysis, physiologically based pharmacokinetic model

## Abstract

Last resort antibiotics, like ceftazidime–avibactam (CZA), were used to treat urinary tract infections caused by multidrug-resistant bacteria. However, no data on tissue distribution were available. Our aim was to describe the *in vivo* kidney distribution of CZA in healthy rats and pigs using a physiologically based pharmacokinetic model (PBPK). Microdialysis probes were inserted into the blood, muscle, and kidney of both species. The experiment started with a retrodialysis by drug period. An i.v. single dose of CZA was administered. Samples were collected for 3 h in rats and 7 h in pigs. A PBPK model was developed to describe tissue and blood CZA pharmacokinetics in animals and to predict human concentrations. The PBPK model adequately described CZA rat and pig data in each tissue and blood. In both species, the concentration profiles of CZA in muscle and blood were almost superimposed, with muscle-to-plasma area under the curve (AUC) ratios close to one. However, kidney CZA concentrations were higher than those in blood, as indicated by kidney-to-plasma AUC ratios exceeding one (respectively 2.27 in rats and 2.63 in pigs for ceftazidime [CAZ]; 2.7 in rats and 4.5 in pigs for avibacam [AVI]). Prediction of human concentrations led to same observations. This study demonstrated an excellent penetration of CZA into the renal parenchyma of rats and pigs. Our PBPK model adequately described the data, and AUCs were higher in the renal cortex interstitium compared with unbound plasma. Our data suggested that the joint PK/PD target for CZA in humans could be attained with reduced CZA doses.

## INTRODUCTION

For more than two decades, health authorities around the world have been concerned about antibiotic resistance. Experts from the WHO predict that in 2050, the incidence of morbidity due to infectious diseases will be similar to that of the 1940s if nothing is done (1).

Urinary tract infections (UTIs), as one of the most common bacterial infections acquired in the community and in hospitals (1), are a major concern. Uropathogenic bacteria are particularly involved in these antibiotic resistance phenomena and are associated with an increasing of deaths caused by infections with antibiotic-resistant bacteria (2).

The severity of antibiotic resistance varies significantly from country to country. For example, some regions in India may have rates exceeding 80% of Enterobacterales producing extended-spectrum beta-lactamases (ESBLs) (3). Similarly, in Europe, resistance rates to third-generation cephalosporins (C3G) and fluoroquinolones in 2022 reached up to 40% for *E. coli* and nearly 80% for *Klebsiella pneumoniae*. Moreover, 12% of *Pseudomonas aeruginosa* strains isolated in invasive infections are carriers of a carbapenemases as suggested by the European Centre for Disease Prevention and Control (https://atlas.ecdc.europa.eu/public/index.aspx). At the same time, antibiotic consumption worldwide continues to increase, leading to a particularly strong antibiotic selection pressure, especially in low socio-economic level countries (4).

In recent years, numerous molecules have been introduced to fight infections caused by multi-drug resistant (MDR) bacteria. These include cefiderocol, meropenem–vaborbactam, ceftazidime–avibactam (CZA), and others. Beta-lactam antibiotics (BLs) combined with beta-lactamase inhibitors (BLIs) are used as last-resort molecules in the treatment of UTIs caused by multi-drug resistant (MDR) bacteria, particularly Enterobacterales producing ESBLs or carbapenemases. Pharmacokinetic and pharmacodynamic (PK/PD) data for these molecules remain incomplete, and all have been derived from plasma data (5), whereas severe UTIs, such as pyelonephritis, are tissue infections. Similarly, urinary tract distribution data solely assess the percentage of the molecule excreted in an active form, independent of its tissue concentration in target organs (5).

Microdialysis is regarded as the gold standard technique in pharmacokinetics for studying the tissue distribution of drugs. Microdialysis is a mini-invasive technique used in daily practice since the 1990s that is composed of a catheter with a semi-permeable membrane, which is perfused with a physiological liquid and introduced in the tissue of choice. This is an *in vivo* sampling technique used to measure endogenous or exogenous molecules present in the extracellular fluid of a specific tissue. During the perfusion, the unbound drug contained in the interstitial space fluid of tissue is filtered out through the semi-permeable membrane by passive diffusion. Because there is no equilibrium on either side of the membrane, the drug (i.e., antibiotic) concentration in the dialysate is proportional to the concentration of the unbound drug (antibiotic) in the interstitial space fluid and can be estimated with microdialysis probe recovery (6). It is a highly potent technique, as it enables continuous sampling from various fluids or tissues within the same animal, providing rich pharmacokinetic (PK) data.

In the context of antibiotic therapy, especially with CZA, the ultimate goal is to optimize treatment effectiveness to reduce UTI-related morbidity and mortality while minimizing the occurrence of toxicity and antibiotic resistance as much as possible. Tissue distribution of antibiotics in humans is challenging to study, and determining it in animals is essential for a better understanding of the PK behavior of these molecules. A better understanding of the distribution of CZA in renal parenchyma would enable the evaluation dosage appropriateness for pyelonephritis treatment based on target site concentrations rather than plasma concentrations. It is especially important in the treatment of MDR bacteria responsible for infections in patients with multiple comorbidities. Considering the epidemiology of pyelonephritis reported in De Lafforest et al. (7), this could potentially represent 10,000 to 20,000 patients per year in France who may require a last-resort antibiotic therapy for this indication.

Developing mathematical PK models enables leveraging the rich PK data set collected by adding microdialysis to plasma sampling to quantify pharmacokinetic behaviour of interest, i.e., the extent of distribution to the kidney cortex. PK models can also include detailed quantitative information about the physiology of the studied species, in which case they are called physiologically based pharmacokinetic models (PBPK). PBPK models are more complex to build than traditional compartmental PK models, but they come with an improved ability to perform inter-species extrapolation when compared with traditional compartmental PK models (8).

Given the difficulty of obtaining these data in humans, we aimed to describe the *in vivo* kidney distribution of CZA in heathy rats and pigs following a single i.v. dose with a PBPK model and to use this model to predict plasma and renal concentrations in humans after a standard dosing regimen and to compare them with the current PK/PD indexes for CZA.

## RESULTS

### *In vivo* recovery

In both species, CAZ and AVI *in vivo* recovery by loss (RL*_in vivo_*) differed between animals for a same medium/tissue and between different media/tissues for a same animal. Probe recoveries were generally lower for CAZ than for AVI in both species, too. In rats, the relative RL*_in vivo_* of both molecules was always lower in the kidney compared with the other tissues that was not the case in pigs. In pigs, relative RL*_in vivo_* are much more variable in a same medium than in rats. All results of mean RL*_in vivo_* are presented Table 1.

**TABLE 1:** *In vivo* mean recovery by loss (RL*_in vivo_*, %) in rats and pigs for ceftazidime (CAZ) and avibactam (AVI) (mean ± SD)

		Mean recovery by loss (%)	
		RAT	PIG
CAZ	Blood	70.6 ± 16.2	78.0 ± 30.0
	Muscle	50.9 ± 5.6	41.3 ± 18.3
	Kidney	44.2 ± 4.7	63.8 ± 22.9
AVI	Blood	80.9 ± 6.7	85.8 ± 25.8
	Muscle	77.6 ± 4.9	47.1 ± 15.5
	Kidney	57.1 ± 13.8	71.2 ± 21.0

### CAZ and AVI transport experiments

CAZ and AVI permeabilities from the apical to basal side (tubule lumen to interstitium) were two times smaller than from the basal to apical (interstitium to tubule lumen) side of LLC-PK1 cells (Table 2).

**TABLE 2:** Apparent *in vitro* permeability of CAZ and AVI across LLC-PK1 cells

	Apparent permeability (cm/s)	
Transport direction	CAZ	AVI
Apical > Basal	1.44 ± 0.46 x 10^−6^	2.53 ± 0.60 x 10^−6^
Basal > Apical	3.10 ± 0.95 x 10^−6^	5.21 ± 1.36 x 10^−6^

### CAZ pharmacokinetics

**Fig 1:**
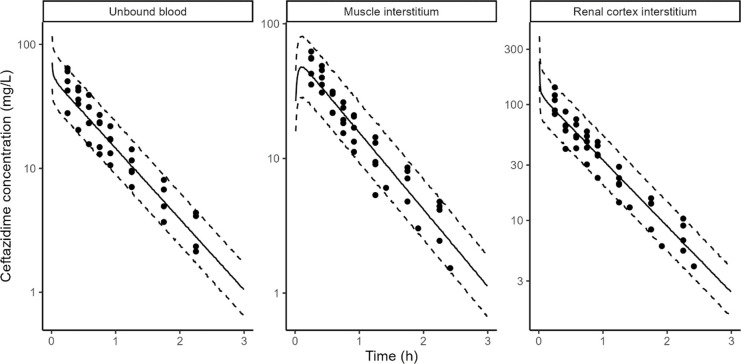
CAZ model fits in rats. Points: observed concentrations, solid line: median of model simulations, dashed lines: 5th and 95th percentiles of model simulations. Prediction interval based on 1,000 simulations. Microdialysis concentrations (points) are shown at midpoint of the collection interval to reflect the fact that they represent average concentrations during the collection interval.

**Fig 2:**
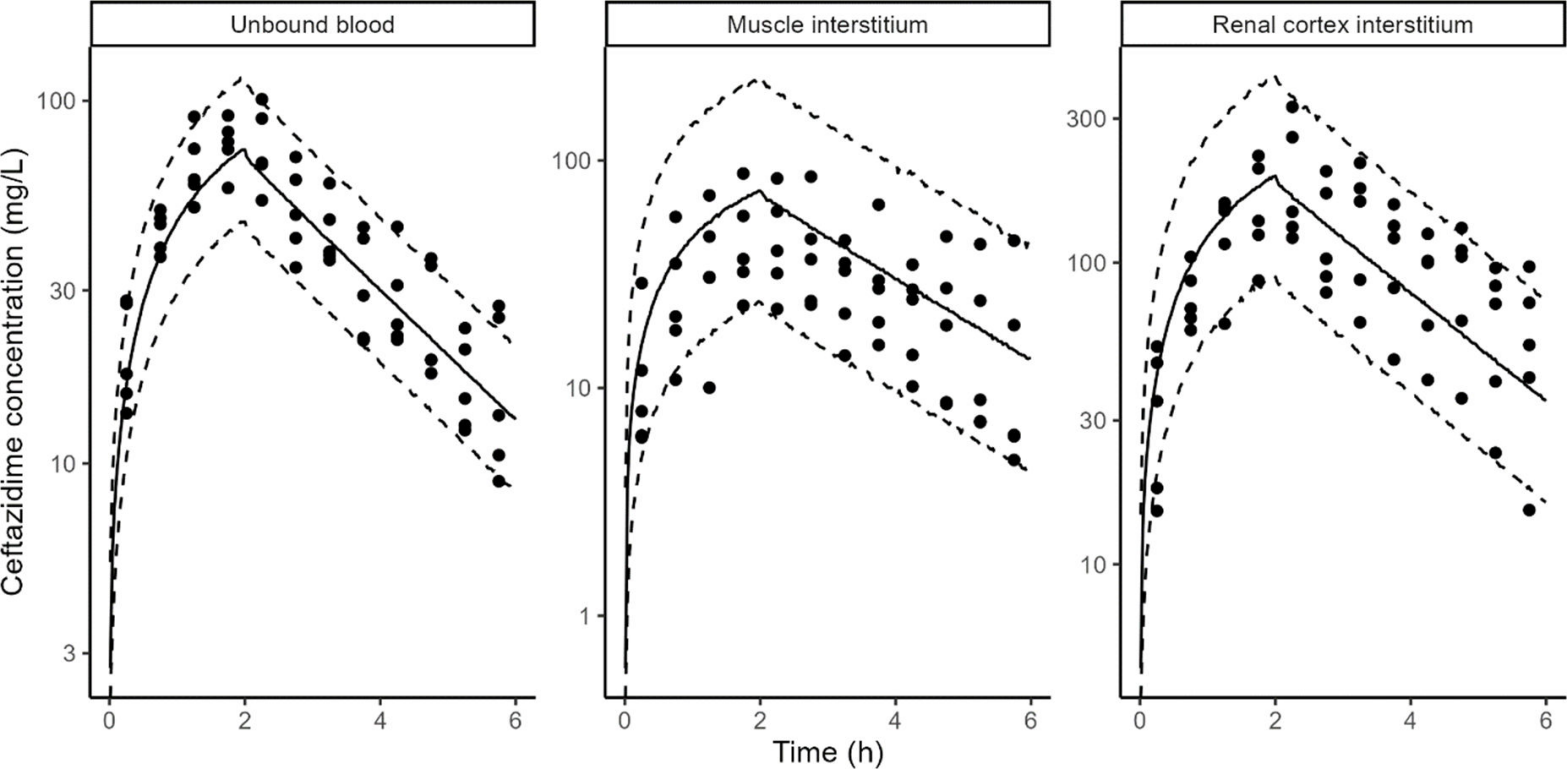
CAZ model fit in pigs. Points: observed concentrations, solid line: median of model simulations, dashed lines: 5th and 95th percentiles of model simulations. Prediction interval based on 1,000 simulations. Microdialysis concentrations (points) are shown at midpoint of the collection interval to reflect the fact that they represent average concentrations during the collection interval.

CAZ rat and pig data in blood, muscle interstitium, and renal cortex interstitium were adequately described by the PBPK model (Fig. 1 and 2).

In addition to glomerular filtration, CAZ was cleared by a non-renal pathway in pigs but not in rats; this non-renal route accounted for 2% of the total clearance (Table 3). The unbound plasma area under the curve (AUC) was identical between muscle interstitium and plasma in both rats and pigs. However, renal cortex interstitium AUC was ~2.5-fold higher than unbound plasma in both rats and pigs AUC, transfer from kidney blood vessels to the kidney interstitium was assumed to be instantaneous (apparent permeability fixed to 100 cm/s), but transfer from the kidney interstitium to kidney blood vessels was fit to the PK data. It was approximately three times lower than the permeability from the blood vessels to interstitium in both rats and pigs (Table 3).

**TABLE 3:** Model-derived CAZ pharmacokinetic parameters^*a*^

Parameter	Rat – median [95% CI]	Pig – median [95% CI]
Total plasma clearance (L/h)	0.154 [0.136–0.179]	6.71 [5.86–8.75]
Renal plasma clearance (L/h)	0.154 [0.136–0.179]	6.41 [5.72–7.19]
Non-renal plasma clearance (L/h)	0 fixed	0.140 [0.0127–2.41]
AUC_muscle interstitium_/AUC_unbound plasma_	1 fixed	1 fixed
AUC_cortex interstitium_/AUC_unbound plasma_	2.27 [2.08–2.47]	2.63 [2.29–2.99]
Apparent permeability from the kidney interstitium to kidney blood vessels (cm/min)	33.8 [30.9–36.9]	31.5 [27.4–36.4]
Apparent permeability from the kidney blood vessels to kidney interstitium (cm/min)	100 fixed	100 fixed

^*a*^ Median of all animals. AUC: area under the curve.

### AVI pharmacokinetics

**Fig 3:**
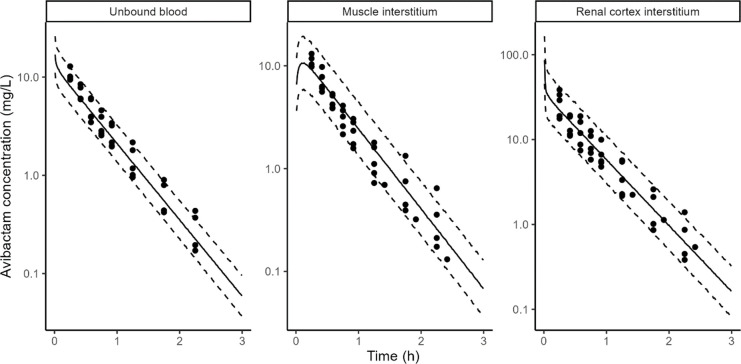
AVI model fit in rats. Points: observed concentrations, solid line: median of model simulations, dashed lines: 5th and 95th percentiles of model simulations. Prediction interval based on 1,000 simulations. Microdialysis concentrations (points) are shown at midpoint of the collection interval to reflect the fact that they represent average concentrations during the collection interval.

**Fig 4:**
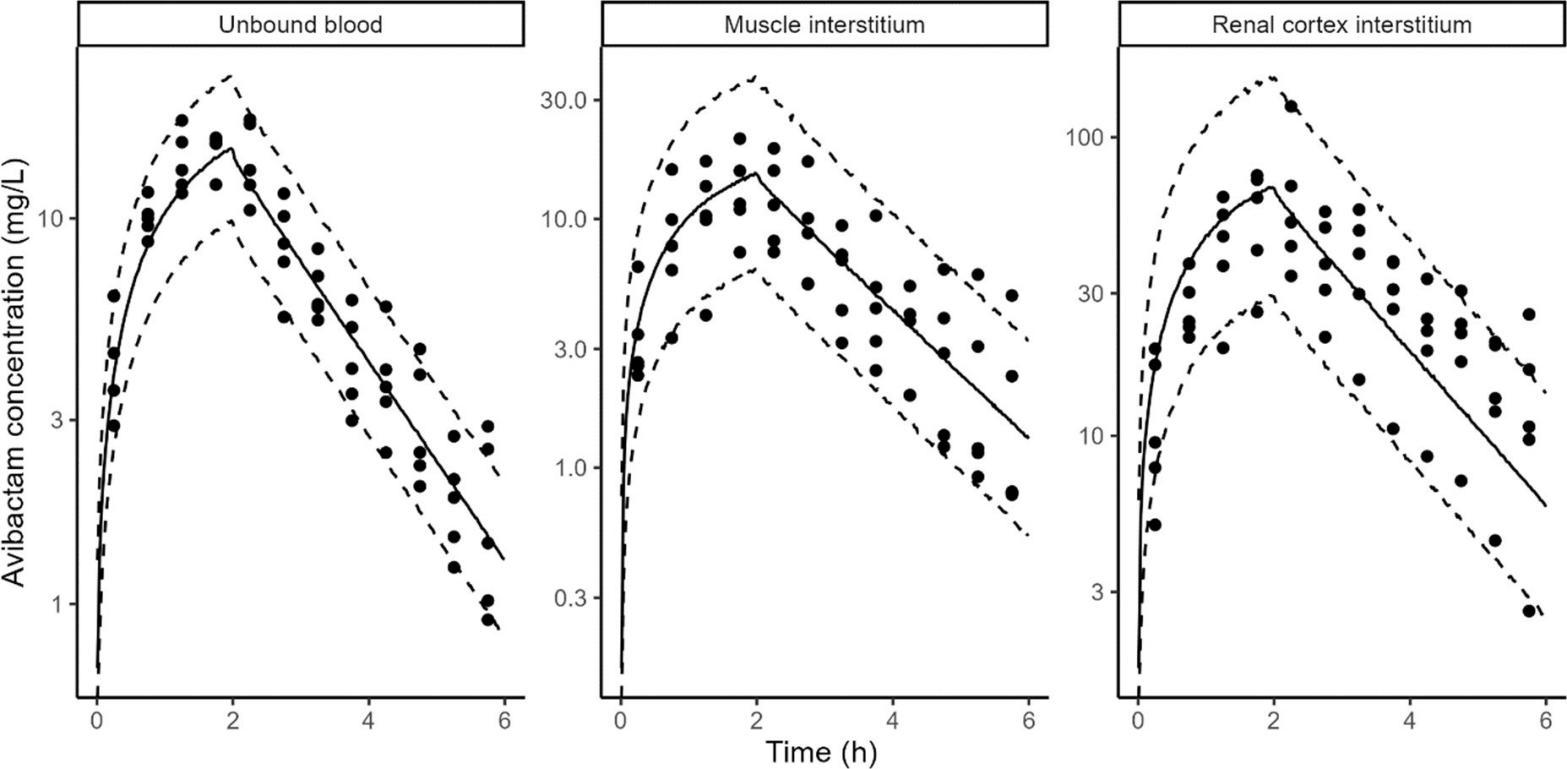
AVI model fit in pigs. Points: observed concentrations, solid line: median of model simulations, dashed lines: 5th and 95th percentiles of model simulations. Prediction interval based on 1,000 simulations. Microdialysis concentrations (points) are shown at midpoint of the collection interval to reflect the fact that they represent average concentrations during the collection interval.

AVI rat and pig data in blood, muscle interstitium, and cortex interstitium were adequately described by the PBPK model (Fig. 3 and 4).

In addition to glomerular filtration, AVI was cleared by tubular secretion in pigs and in rats, this secretion accounted for 27% (rats) and 18% (pigs) of the total clearance (Table 4). The unbound plasma area under the curve (AUC) was identical between muscle interstitium and unbound plasma in both rats and pigs. However, renal cortex interstitium AUC was ~2.7- (rats) and ~4.5- (pigs) fold higher than unbound plasma. Transfer from kidney blood vessels to the kidney interstitium was assumed to be instantaneous (apparent permeability fixed to 100 cm/s), but transfer from kidney interstitium to kidney blood vessels was fit to the PK data. It was approximately six lower than the permeability from the blood vessels to interstitium in both rats and pigs (Table 4).

**TABLE 4:** Model-derived AVI pharmacokinetic parameters^*a*^

Parameter	Rat – median [95% CI]	Pig – median [95% CI]
Total plasma clearance (L/h)	0.220 [0.191–0.256]	9.78 [8.76–10.9]
Renal plasma clearance (L/h)	0.220 [0.191–0.256]	9.78 [8.76–10.9]
Glomerular plasma clearance (L/h)	0.158 [0.139–0.256]	5.71 [5.11–6.49]
Tubular secretion plasma clearance (L/h)	0.0612 [0.0458–0.0786]	3.96 [3.39–4.64]
AUC_muscle interstitium_/AUC_unbound plasma_	1 fixed	1 fixed
AUC_cortex interstitium_/AUC_unbound plasma_	2.70 [2.39–3.09]	4.50 [3.95–5.10]
Apparent permeability from the kidney interstitium to kidney blood vessels (cm/s)	18.5 [15.6–22.2]	16.4 [14.4–18.8]
Apparent permeability from the kidney blood vessels to kidney interstitium (cm/s)	100 fixed	100 fixed

^*a*^ Median of all animals. AUC: area under the curve.

### PK simulations in humans

**Fig 5:**
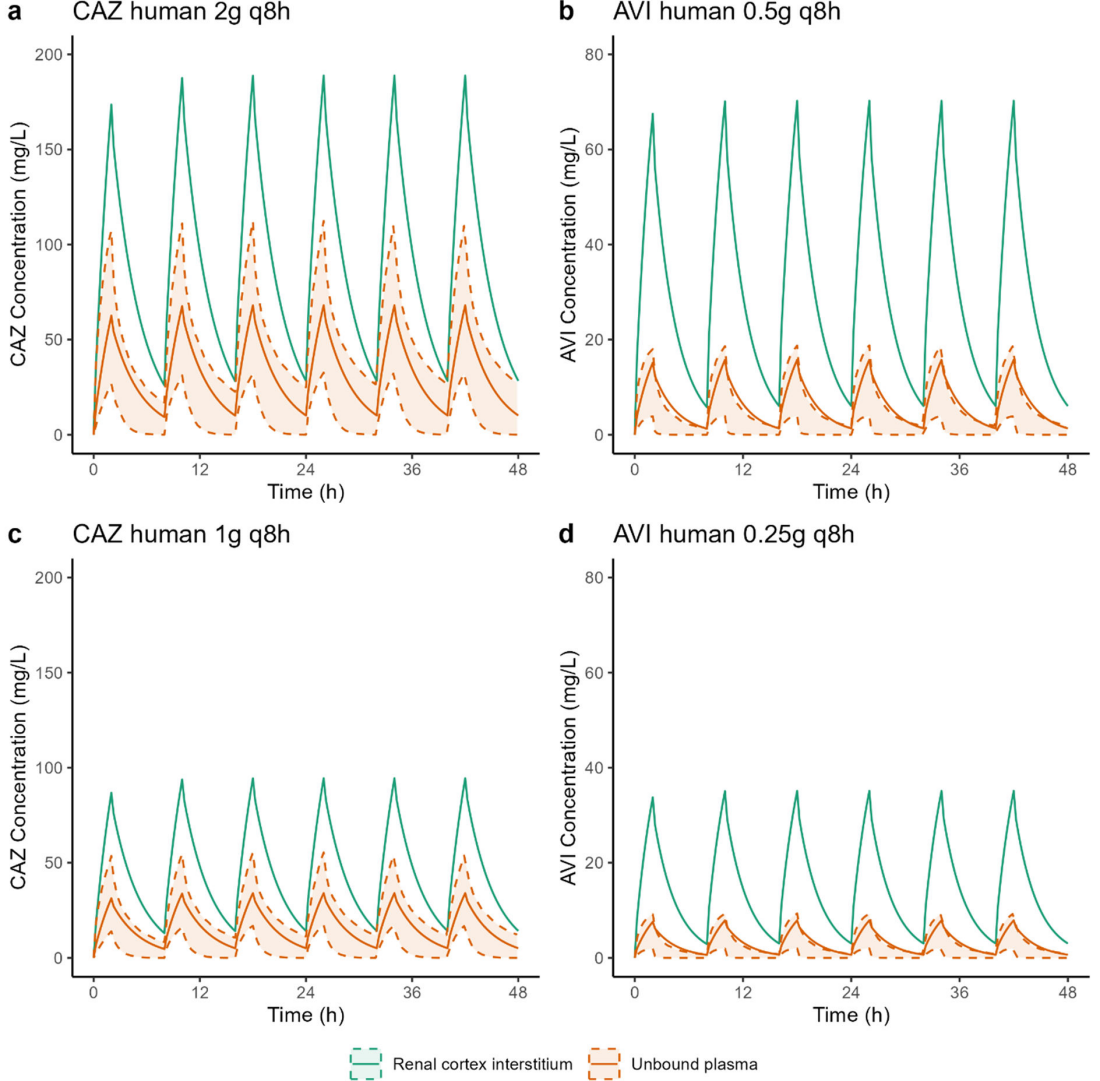
Simulated unbound CAZ (**a and c**) and AVI (**b and d**) concentrations in human plasma (orange line) and renal cortex interstitium (green line) following the standard CZA dosing regimens of 2 g/0.5 g q8 h (upper panels) and a reduced regimen of 1 g/0.25 g q8 h (lower panels). The orange shaded area represents the 90% CI of 1,000 simulations performed with the Li et al. (9) population PK model.

Using the PBPK model, simulations of unbound CAZ and AVI concentrations in the plasma and renal cortex interstitium were conducted for a healthy adult following the standard CZA dosing regimens 2 g/0.5 g q8 h (Fig. 5a and b) as well as at half the dose (Fig. 5c and d). The simulated steady-state maximal concentrations of CAZ and AVI in the human renal interstitium were 2.76 and 4.46 times higher, respectively, than those in unbound plasma for both dosing regimens (Fig. 5).

**Fig 6:**
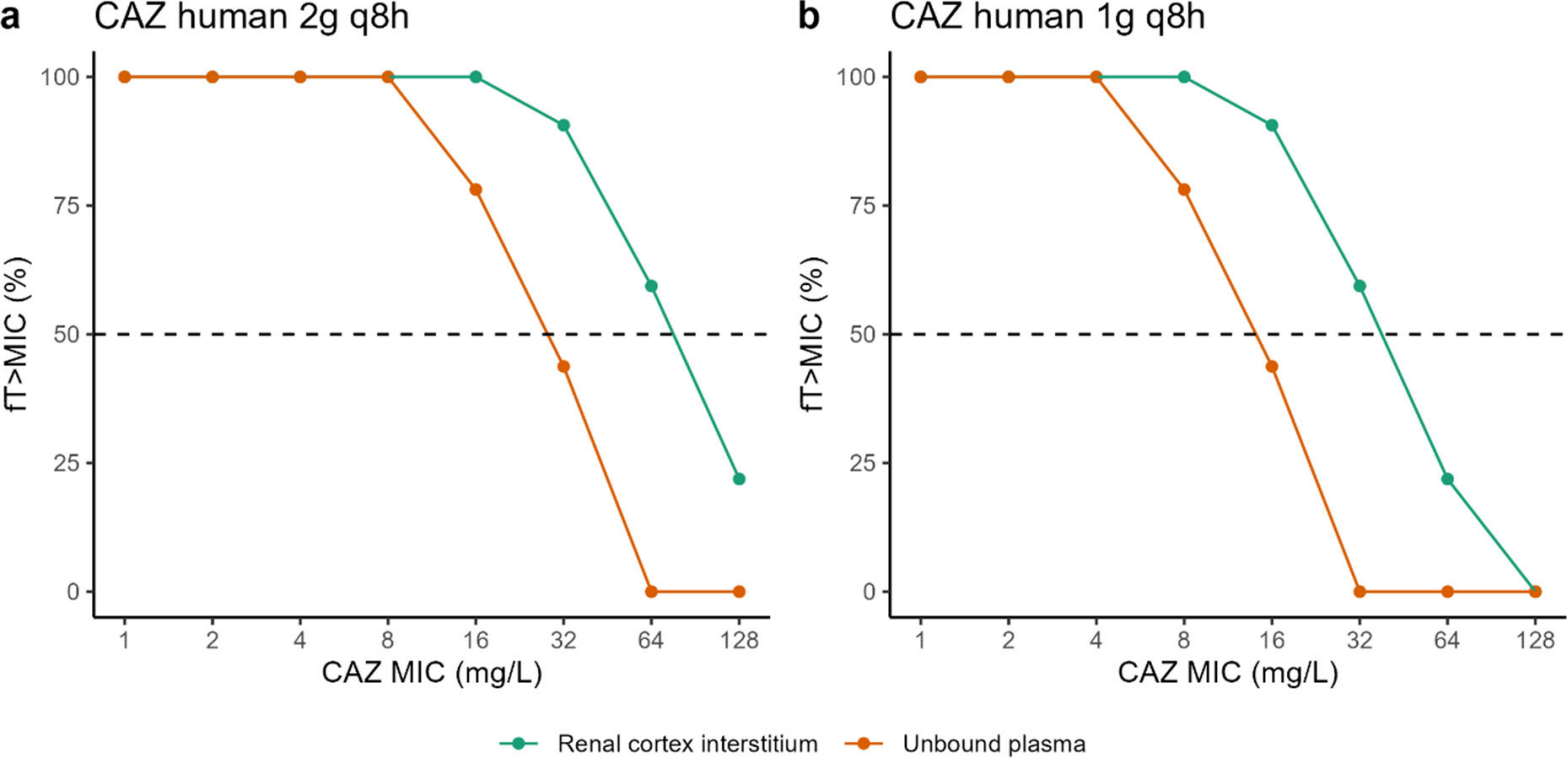
Proportion of the dosing interval during which the unbound CAZ concentration in the plasma and renal cortex exceeds the target bacteria’s MIC for both current (2 g q8 h) (**a**) and reduced (1 g q8 h) (**b**) dosing regimens. Dashed line represents 50% fT > MIC the current EUCAST target for CAZ (10).

With the standard dosing regimen, 50% of free time above the MIC can be achieved for MICs fourfold higher in the renal interstitium compared with unbound plasma (Fig. 6a). With the reduced dosing regimen, %fT > MIC = 50% is achieved in the renal interstitium for MICs up to 32 mg/L (Fig. 6b), while the target was not achieved in unbound plasma with the standard dose (Fig. 6a).

Moreover, for both dosing regimens, the PK/PD target for AVI of 50% *f*T > C_T_ (target concentration) of 1 mg/L MIC (10) was always achieved (100% for both dosing regimens) (Fig. 5b and d).

## DISCUSSION

This study demonstrates the excellent penetration of CZA into the renal parenchyma, with tissue concentrations, which may significantly surpass the usual MICs of bacteria responsible for acute pyelonephritis, including MDR bacteria. These findings enhance the utility of this combination in treating acute pyelonephritis. It has already been established that this combination is almost completely excreted in the urine (94.9% to 99.6%), reaching effective target concentrations at standard doses (11). The results obtained within the renal parenchyma across two different species are novel and confirm the theoretical appeal of this BL–BLI combination based on the achieved concentrations.

Microdialysis is a preferred tissue sampling technique for conducting tissue pharmacokinetic studies with rich data. However, in kidney studies, this technique has been preferentially used in models of organ preservation and transplantation to assess organ viability before and after transplantation by collecting endogenous markers of tissue health either in *ex vivo* models (12–14) or in rats (15). Regarding the measurement of exogenous drugs by microdialysis, only a few studies have been carried out, and the use of rats remains the conventional model (16–21). All the microdialysis studies carried out to assess the renal distribution of anti-infective agents, such as antifungals, were conducted by Dalla Costa et al. in healthy or infected rats (17–19).

In the present study, we described renal pharmacokinetics of CZA in both rats and pigs, allowing interspecies extrapolation. The microdialysis probe was inserted within the extracellular space of kidney cortex (composed of the peritubular interstitium and extraglomerular mesangium), constituting a minor portion of the renal cortex (22). The cortical area of the kidney is particularly affected during pyelonephritis, as evidenced by radiological studies showing the predominance of lesions in this part of the renal parenchyma, forming the classic triangular area of hypoattenuation (23).

Although, microdialysis is being praised for its minimal tissue damage and its non-interference with biological fluid measurements (24, 25), insertion of a probe into the renal cortex could potentially affect the surrounding glomeruli. We attempted to evaluate this by examining the probe insertion area within the renal parenchyma using optical microscopy post-procedure. Unfortunately, due to the small size of the probe, it was not possible to isolate the probe insertion area on the slides. Consequently, we cannot confirm or refute the atraumatic nature of the probe placement. Nonetheless, the microscopic analysis of the porcine renal parenchyma enabled us to confirm its histological similarity to human renal parenchyma, which has also been demonstrated in previous studies (26). Several studies employing microdialysis in both the cortex and medulla consistently found higher concentrations of endogenous molecules (lactates, pyruvate...) in the medulla than in the cortex, suggesting different metabolic processes (14, 27, 28). For renal distribution studies of exogenous compounds, some teams inserted the microdialysis probe into the medulla without clear rationale (29). Consequently, we carried out preliminary tests on pigs with the probe placed both in the cortex and renal medulla. Correct positioning of the probes was confirmed by ultrasound. The results were consistent for both probes, leading us to simplify the procedure by retaining only the cortical probe for easier placement. A microdialysis probe was also inserted into muscle, and in the absence of barriers and transporters, only passive diffusion drives exchange, making muscle the control tissue of choice, where exposure to antibiotic should not differ significantly from that in blood.

One of the challenges of the microdialysis technique is the estimation of probe recoveries. In the present study, the *in vivo* retrodialysis by loss of the drug method was used in both species before PK experiments (6). It is generally assumed that a recovery above 20% provides a good approximation of the actual concentration at a given time (6), which was the case in the present study where all our recoveries were above 40% (Table 1). This recovery depends on a number of factors, including the molecule, its environment, perfusion rate, and membrane length (6). In this study, probe recoveries were generally lower for CAZ than for AVI in both species because AVI has a lower molecular weight than CAZ. Moreover, in rats, the relative RL*_in vivo_* of both molecules was always lower in the kidney compared with the other tissues due to the smaller length of microdialysis membrane used in this tissue, which was not the case in pigs.

Our mechanistic kidney PBPK model is an adaptation of previous works by Li et al., Huang and Isoherranen, and Scotcher et al. (30–32). The main novelty is the differentiation of the interstitial and blood compartments, which was not included in any previous work. This separation was necessary to be able to describe the observed differences between unbound plasma and cortical interstitial concentrations, which is incompatible with the standard assumption of instantaneous equilibrium between plasma and interstitium. This relaxation of the instantaneous equilibrium assumption is modelled by a slower transfer from the kidney interstitium to kidney blood than from kidney blood to the kidney interstitium (Tables 3 and 4). The mechanistic reason for this slower transfer rate is unknown. Limited data about kidney arterioles permeability to drugs were found in the literature, with most papers focusing on small ions and their reabsorption (33). Data on cortical interstitium permeability to drugs are also lacking. Further histological and physiological studies would be necessary to understand the mechanism of the high CAZ and AVI concentrations observed in cortical interstitium, but these are beyond the scope of the present study. However, data from the literature show similar observations. Indeed, some researchers have investigated the diffusion of ceftazidime within the renal parenchyma by measuring concentrations in *ex vivo* tissue homogenates after the rats’ sacrifice (34, 35). Acknowledging the pharmacokinetic limitations of the homogenate method, which cannot differentiate intra- and extracellular concentrations (36), it is noteworthy that these results align with ours, showing high renal tissue concentrations exceeding those in plasma. Moreover, Eickenberg et al. studied interstitial concentrations of antibiotics in the kidney using “tissue cages,” which are multiperforated polypropylene balls implanted in renal parenchyma and sampled after a set time for concentration determination. They demonstrated that the recovered fluid was similar to interstitial fluid (37). In a dog study of renal penetration of three cephalosporins (cefamandole, cephalothin, and cefazolin), they found renal interstitial fluid concentrations higher than plasma concentrations for all three drugs (38), adding confidence to our data. In rats and pigs, we found that AVI was renally secreted in addition to being filtred with secretion clearances representing 27% and 18% of total clearance in rats and pigs, respectively. This is in line with human data since AVI was reported to be substrate of the OAT1 and OAT3 transporters, and secretion clearance was 20% of the total AVI clearance (39).

The PBPK model was used to simulate the plasma and renal concentrations of CAZ in healthy adult at two different dosing regimens (Fig. 5). The simulated unbound plasma concentrations of CAZ are consistent with literature data (9), while AVI unbound plasma concentrations are in the upper range of expected concentrations, indicating a potential overestimation of AVI concentrations with our PBPK model; however, the predicted concentrations remain in the right order of magnitude. Overall, this comparison provides confidence in the preclinical data generated in rats and pigs and the developed PBPK model. It is important to recognize that the PK/PD targets for the kidney may also differ from those for plasma, which could influence the efficacy of the two drugs accordingly. Further research should be carried out to determine specific exposure targets in the kidney. But, considering a joint PK/PD target of 50% *f*T > MIC for CAZ and 50% *f*T > C_T_ of 1 mg/L for AVI, thus assuming that the PK/PD target is the same in the cortex interstitium and plasma, the results of the simulations (Fig. 6) suggest that the standard dose of CZA of 2 g/0.5 q8 h could be used to treat renal infections due to bacteria with MICs four times higher than the breakpoint, or the dose of CZA could be halved, thus limiting the medico-economic costs of these last-resort treatments, which are very high compared with daily use antibiotics (40). Of course, we must remain cautious, and these results highlight the need for confirmation in a larger prospective clinical study, which has not yet been initiated. Investigating the effect of infection on CZA distribution in an infected pig model would be highly beneficial. However, developing such a model presents challenges, including the lack of standardized protocols and the need to evaluate its translational relevance.

This study demonstrates the excellent penetration of CZA into the renal parenchyma in rats and pigs. The developed PBPK model successfully described tissue data, confirmed by higher AUCs in the renal cortex interstitium compared with unbound blood in both species. This model was then used to extrapolate these animal data to humans to evaluate therapeutic regimens.

## MATERIALS AND METHODS

### Chemicals

Ceftazidime–avibactam (CZA) (ZAVICEFTA 2 g/0.5 g, powder for dilution for intravenous infusion; Pfizer Laboratories, New York, USA) was used to prepare CZA solutions in 0.9% NaCl or Ringer solution for intravenous (i.v.) administration and probe perfusion, respectively. For assay, avibactam and ceftazidime standard (purity of 99.9% and 88.5% in powder form) was purchased from MCE (Monmouth Junction, USA) and Sigma Aldrich (Saint Louis, USA), respectively. [^13^C_5_] Avibactam and [^2^H_6_] ceftazidime (purity of 100% and 98.1% in powder form) were obtained from Alsachim (Strabourg, France). High-performance liquid chromatography (HPLC) grade acetonitrile was obtained from Fisher Scientific (Hampton, USA).

### Animals

Experiments in rats and in pigs were carried out in accordance with EC Directive 2010/63/EU. They were approved by the local ethics committees (COMETHEA and CEEA VdL committee number 19) and respectively registered by the French Ministry of Higher Education and Research (Authorization number APAFIS#29408–2021012917483163 v4 and APAFIS#38762–2022122015407214 v2). Five, 10-week-old, male Sprague Dawley rats from Janvier Labs (Saint Berthevin les Laval, France) weighing between 300 and 350 g and five male Large White pigs from UE PAO, piggery (Nouzilly, France) aged 2–3.5 months old and weighing between 24.7 and 52.3 kg were used. All rats were acclimatized in ventilated rack in temperature regulated environment with a 12 h light-dark cycle, with also free access to food and water for a minimum of 5 days before the beginning of the experiment. Each pig was transported to the laboratory on the same day as the experiment.

### Surgery: catheter, vein, muscle, and kidney probe insertion

#### In rats

The day before the experiment, rats received subcutaneous administration (SC) of buprenorphine (Buprecare, Axience, Pantin, France) at a dose of 0.05 mg/kg before being anesthetized by isoflurane (Forene, Abbot, Rungis, France) inhalation (5% in inhalation chamber followed by 2% under mask). Surgical sites were meticulously prepared, involving shaving and cleaning/disinfection, using Vetedin soap (Vetoquinol, Lure, France) followed by the solution (Vetoquinol, Lure, France). Polyethylene cannula was then inserted into the left femoral vein for drug administration as previously described (41). Two CMA/20 probes (polyarylethersulfone [PAES]; cutoff, 20,000 Da; membrane length, 10 mm; CMA microdialysis; Phymep, Paris, France) were inserted into the right jugular vein and the right hind leg muscle as previously described (41). Briefly, the first probe, perfused (1 µL/min) with 1% low-molecular-weight heparin (Lovenox, Sanofi-Aventis, France) was inserted through the pectoral muscle into the right jugular vein and then secured by suturing it to the pectoral muscle. The second one, perfused (1 µL/min) with Ringer solution (perfusion fluid T1 for peripheral tissue; CMA microdialysis; Phymep, Paris, France) was inserted into the right hind leg muscle. After their insertion, the two probes were flushed at 10 µL/min for 10 min. The inlet and the outlet of probes as the femoral catheter were passed subcutaneously to exit at the nape. Animals were awakened in individual cages. On the day of experiment, rats were re-anesthetized by isoflurane inhalation and put in dorsal position. A 2 cm incision was made under the ribs along a vertical axis traced between the right ear and tail. The kidney was isolated from the fat and a CMA/20 probe (PAES; cutoff, 20,000 Da; membrane length, 4 mm; CMA microdialysis; Phymep, Paris, France), perfused with Ringer, was inserted into the renal cortex with the help of an introducer. The probe was sutured to the abdominal muscle and the skin was closed.

#### In pigs

The day of experiment, the induction of anesthesia was made by an IM injection of a ketamine (20 mg/kg) and xylazine (2 mg/kg) mixture (Imalgène 1000 [Merial, Lyon, France]) and Rompun 2%, (Elanco AH, Cuxhaven, Germany) followed by isoflurane inhalation at 5% through a mask in spontaneous mode using Dräger Tiro devices. An intramuscular injection of Buprecare (0.05 mg/kg) was administered, followed by the application of lidocaine spray (Xylocaine spray, Aspen, Rueil Malmaison, France) to the throat. Endotracheal intubation was then performed utilizing Xylocaine Visqueuse 2% (Aspen, Rueil Malmaison, France). Subsequently, the pig was carefully positioned on the surgical table in the lateral right decubitus position. Catheters of 24G or 22G (BD Insyte UU, BD Medical, France) were placed in each auricular vein for intravenous perfusion purposes. The isoflurane concentration was adjusted to 2% through the intubation probe, and controlled ventilation (10 mL/kg, temperature, SaO_2_, EtCO_2_, cardiac frequency) was initiated. Intravenous perfusions with Ringer Lactate (800 mL/h) and Glucose 5% (40 à 80 mL/h) were established. The surgical site was meticulously prepared, involving shaving and disinfection using Vetedin soap, followed by the solution (Vetoquinol, Lure, France). Local anesthesia was administered subcutaneously using 1 to 2 mL of Lurocaine (Vétoquinol, Lure, France) in each surgical area before incision. Following the initial preparations, an arterial catheter (Vygon, Ecouen, France) was percutaneously inserted, guided by ultrasonography, into the brachial artery of the front right leg (Fig. S1a). Microdialysis probes (CMA 63, polyarylethersulphone membrane; cutoff, 20,000 Da; membrane length, 10 mm; 4Med; Serris, France) were delicately inserted into the cephalic vein of the front right leg guided by ultrasonography, and into the left longissimus dorsi.

The procedure continued with the insertion of a third CMA 63 probe into the cortex part of the left kidney via open retroperitoneal surgery. Intraoperative ultrasonography confirmed the probe’s accurate positioning, and it was secured to the kidney using two sutures of 5.0 Prolene.

Three hours after the start of the procedure, a suprapubic catheter was guided by ultrasonography and inserted into the bladder to monitor diuresis and adjust the quantity of Ringer Lactate perfusion.

### Pharmacokinetic study

#### *In vivo* recovery calculations in rats and pigs

The perfusion flow rate was maintained to 0.5 µL/min for all the duration of experiment. The pharmacokinetic experiment started with a retrodialysis by drug period, during which all probes were perfused with Ringer containing CZA (100 µg/mL/10 µg/mL and 50 µg/mL/12.5 µg/m for CAZ and AVI in rats and pigs, respectively) to determine the *in vivo* recovery by loss of probes. After an equilibration period of at least 45 min, three microdialysate samples were collected for 60 min by fractions corresponding to 20 min intervals. A washout period of at least 1 h with blank Ringer solution perfusion was allowed before i.v. CZA administration.

To determine the *in vivo* recovery by loss (RL*_in vivo_*), the CAZ and AVI concentrations in the perfusate (*C*_in_) and in dialysates (*C*_out_) were determined by liquid chromatography–tandem mass spectrometry (LC-MS/MS). The RL*_in vivo_* was expressed as a percentage and was calculated for each interval of time as follows: RL*_in vivo_* = [(*C*_in_ - *C*_out_)/*C*_in_] × 100. The *in vivo* recovery used to correct the dialysate concentrations was the mean value obtained from the three individual determinations.

#### CZA administration

An i.v. single dose of CZA (respectively, 20 mg/kg of CAZ and 5 mg/kg of AVI as an i.v bolus in rats and 40 mg/kg of CAZ and 10 mg/kg of AVI as a 2 h infusion in pigs) was administered.

#### Microdialysis experiment

In rats, microdialysis samples were taken every 10 min for 1 h, then every half-hour for 1.5 h. In pigs, microdialysis samples were taken every 30 min for 6 h. All samples were stored at −80°C until LC-MS/MS assays.

#### Microdialysis sample analysis

CAZ and AVI assays in dialysates from rat or pig were performed by an adaptation of LC-MS/MS methods, previously developed for the quantification of these molecules in rat samples (42). Calibration curves were established in Ringer over 0.05 to 50 µL/min for both compounds. Directly after collection, microdialysates were diluted (1:5 [vol/vol]) with acetonitrile with 0.1 formic acid solution spiked with both internal standard [^13^C_5_] avibactam at 0.5 µL/min and [^2^H_6_] ceftazidime at 0.25 µL/min and were directly injected onto LC-MS/MS. The system included a Shimadzu high-performance liquid chromatography system module (Nexera XR; Shimadzu, Marne la Vallée, France) coupled with a TQ 3500 mass spectrometer (Sciex, Les Ulis, France). Both compounds were analyzed on an Excel 3 C18 AR column (3 µm, 50 by 4.6 mm [inside diameter]; ACE, UK). The mobile phase A consisted of water with 0.1% formic acid, and mobile phase B was acetonitrile with 0.1% formic acid. The gradient elution program started with 10% of mobile phase B, which was maintained for 0.5 min, then ramped to 40% mobile phase B by 2 min and then ramped again to 95% by 0.5 min that composition was maintained until 1 min. The mobile phase composition was then reverted to 10% mobile phase B at 4 min and that was maintained until the end of the chromatographic run at 6 min. Electrospray ionization in both negative and positive modes were used for the detection of AVI and CAZ, respectively. Ions were analyzed in the multiple reaction monitoring, and the following transitions were inspected: m/z 264→96.2 for AVI, m/z 269→95.8 for its labeled internal standard, and m/z 547.9→469.0 for CAZ and m/z 553→474 for the CAZ-labeled internal standard. The intraday variability was characterized at three concentration levels (30, 3, and 0.3 µg·mL^−1^ for both compounds) with a precision and bias of <15% for both compounds (*n* = 6 per molecule and per matrix). Corresponding between-day variability was determined with a precision and a bias of <15% (*n* = 18 per molecule and per matrix).

### LLC-PK1 transport experiments

#### Cell culture

Pig kidney LLC-PK1 cells were purchased from American Type Culture Collection (ATCC CL-101 – lot number: 64143751) and grown in medium 199 supplemented by 2 g/L sodium bicarbonate, 50 units/mL penicillin, 50 µg/mL streptomycin, and 3% fetal bovine serum. The atmosphere was kept at 37°C, 90% to 95% relative humidity, and 5% CO_2_ in air. The cells were used at passages 5–10 for all experiments. For routine culture, LLC-PK1 cells were plated in cell culture dishes and subcultured before reaching confluency using a trypsin-EDTA (0.25%) solution. The culture medium was renewed twice a week.

For transport experiments through barrier models, LLC-PK1 was seeded at a density of 5 × 10^5^ cells/cm^2^ on top of 24-well semipermeable Transwell inserts (PET membrane, 0.33 cm² with pore sizes of 0.4 µm and pore density of 2.0 ± 0.2 × 10^6^/cm²). For experiment culture, LLC-PK1 cells were maintained in medium 199 supplemented by 2 g/L sodium bicarbonate and 3% fetal bovine serum.

Prior to seeding the cells, the insert filters were pre-coated with 6.1 µg/cm² rat-tail collagen type I. Cells were then cultured 4 days in liquid–liquid interface (0.3 mL in apical [AP] compartment and 0.9 mL basolateral [BL] compartments) to allow formation of tight junctions in the LLC-PK1 monolayer. Its integrity was assessed by measuring transepithelial electrical resistance (TEER) with a Millicell ERS-2 Voltohmmeter. In order to reduce temperature-dependent variability, cultures were equilibrated at room temperature in HBSS for 15 min before resistance measurements. To calculate the TEER values for each LLC-PK1 monolayer, the TEER value of cell-free coated insert was subtracted from the TEER value obtained in the presence of cells. These values were multiplied by the effective surface area of the filter (0.33 cm^2^), and the final values were expressed as Ω·cm^2^. We thus ensured that all transport experiments were performed on well-established barriers with a TEER of at least 100 Ω.cm^2^.

#### CAZ and AVI transport experiments

Transport experiments were conducted in both AP-to-BL and BL-to-AP directions. On the study day, after verification of the barrier integrity, the LLC-PK1 monolayers were incubated for 15 min in HBSS. Following this equilibration period, the apical or basolateral media (for AP-to-BL and BL-to-AP transports respectively) were replaced by HBSS containing 100 mg/L of CAZ and/or 25 mg/L of AVI, and the plates were returned to the incubator. Subsequently, sample aliquots were taken from the BL or AP compartments (for AP-to-BL and BL-to-AP transports, respectively) at 3, 4.5 and 6 h. Following the transport study, a control of the monolayer integrity was performed by TEER measurement as previously described. Two independent experiments were performed.

### PBPK modeling

#### Modeling of animal data

**Fig 7:**
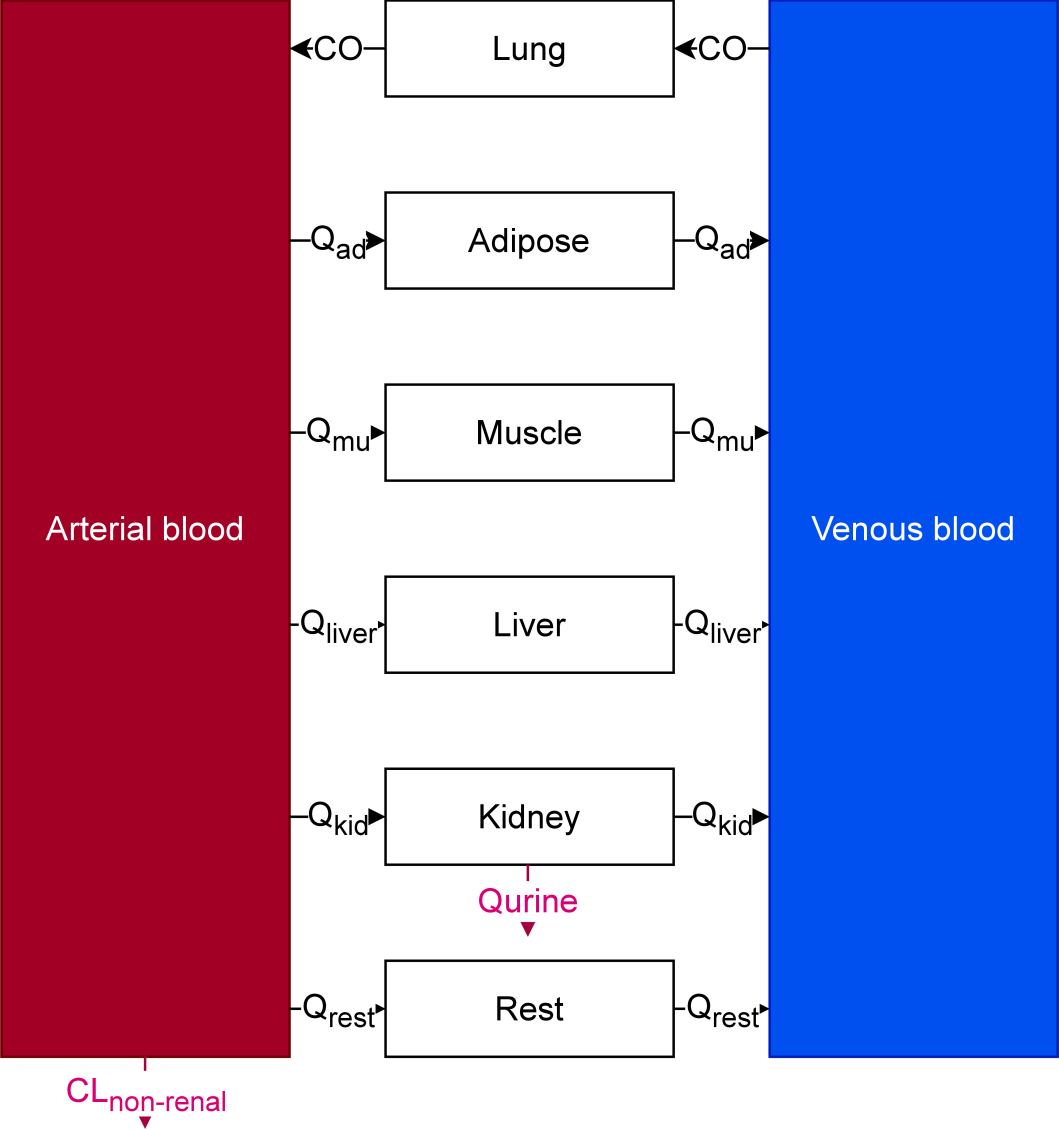
General structure of the PBPK model. CO: cardiac output, Q_ad_: blood flow to adipose tissue, Q_mu_: blood flow to muscles, Q_liver_: blood flow to liver, Q_kid_: blood flow to kidneys, Q_rest_: blood flow to the rest of body compartment, Q_urine_: urinary flow, CL_non-renal_: non renal clearance.

**Fig 8:**
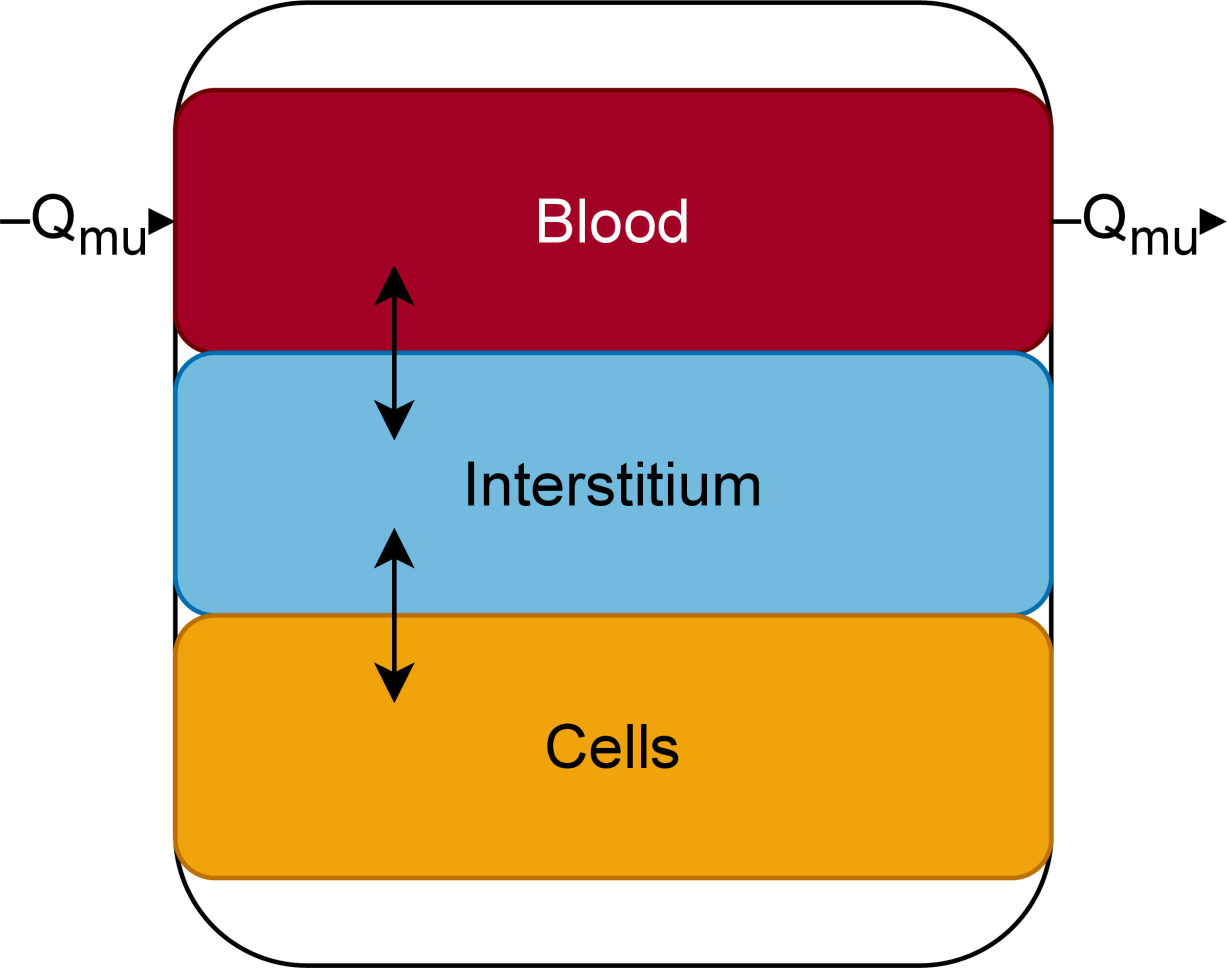
Detailed structure of the muscle compartment. Q_mu_: blood flow to muscles.

**Fig 9:**
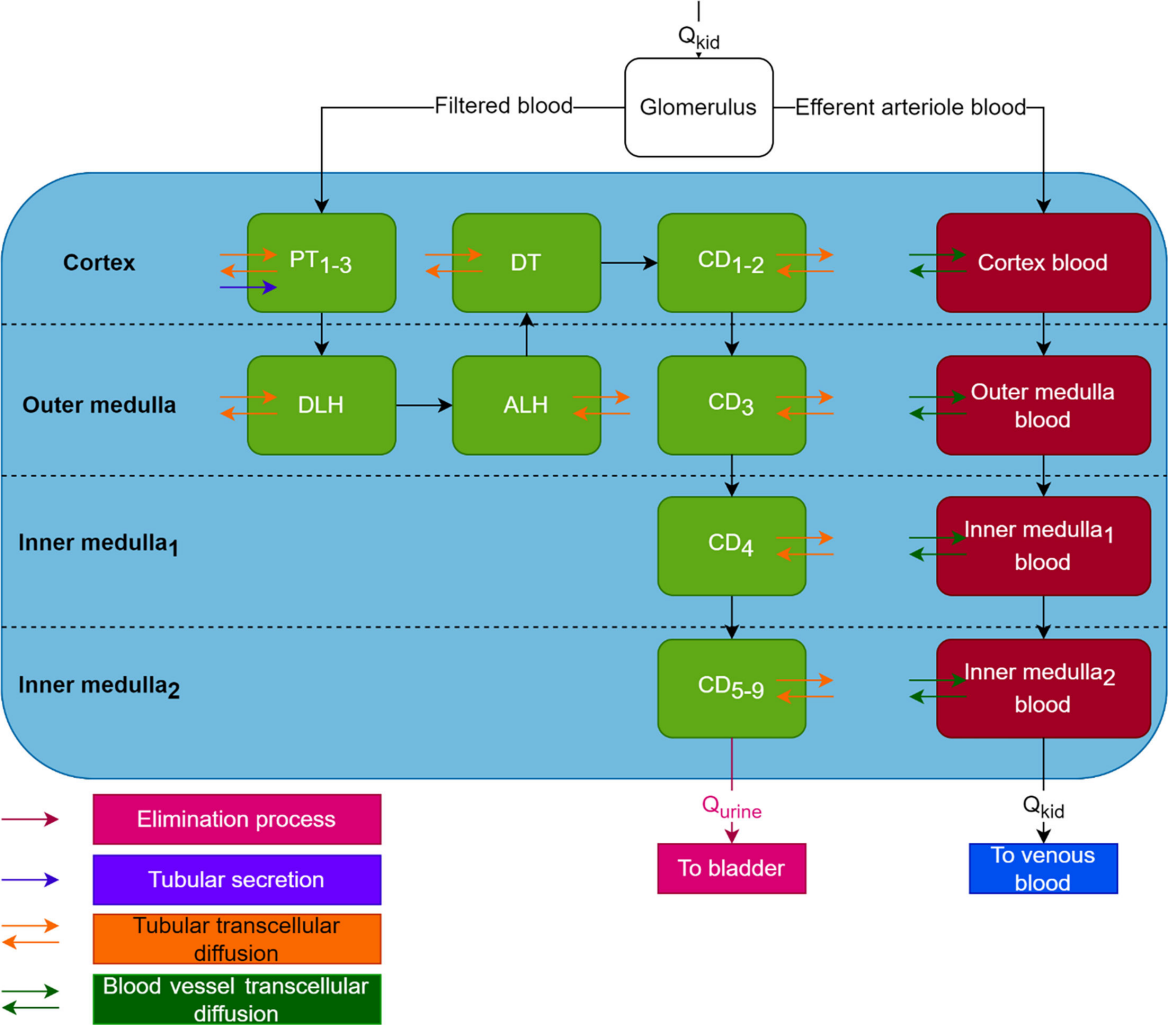
Detailed structure of the kidney compartment. Q_kid_: blood flow to kidneys, Q_urine_: urinary flow, PT: proximal tubule, DLH: descending loop of Henlé, ALH: ascending loop of Henlé, DT: distal tubule, CD: collecting duct. Green: tubular lumen compartments, light blue: interstitium compartments, red: blood vessel compartments.

The general structure of the PBPK model is found on Fig. 7. Blood was split into two compartments, venous and arterial blood. Lungs, adipose tissue, and liver were represented as homogenous compartments to which drug distribution would be perfusion limited (i.e., it was assumed that any molecule that reaches the compartment instaneously distributes to the whole organ). Muscles were split into three subcompartments representing vascular, interstitial, and cellular spaces. Thus, drug distribution was permeability limited. Its structure will be further described below (Fig. 8). Kidneys were split into four anatomical compartments which were further split into subcompartments. Its structure will be further described below (Fig. 9).

The muscle compartment was split into three subcompartments representing the vascular, interstitial, and cellular spaces with passive transfer between all three spaces (Fig. 8).

The kidney compartment model was adapted from Li et al*.,* Huang and Isoherranen, and Scotcher et al. (30–32) and represented on Fig. 9. The kidney was split into four anatomical compartments, the cortex, outer medulla, and two inner medulla compartments. Each compartment was split into subcompartments representing tubules (green compartments on Fig. 9), interstitial space (light blue compartments on Fig. 9), and blood vessels (red compartments on Fig. 9). The proximal tubule was split into three subcompartments (PT_1-3_) (31). The collecting duct was split into nine subcompartments, one compartment for each nephron fusion event (CD_1-9_) (30). Transcellular permeability across tubular cells was fixed to the *in vitro* apparent permeability values measured on LLC-PK1 cells (orange arrows on Fig. 9). Transcellular permeability across blood vessels (dark green arrows on Fig. 9) from interstitium to blood was fit to data, transcellular permeability across blood vessels from blood to interstitium was fixed to a high value (100 cm/s).

Model fitting was performed using Monolix (Monolix 2024R1, Lixoft SAS, a Simulations Plus company). Details about the model parameters and equations are given in the supplementary material.

#### Computation of PK parameters

Simulations under the final model of the PK profiles from 0 to 100 h (arbitrary time at which the drugs are fully eliminated) were performed for each animal included in the study with Simulx (Simulx 2024R1, Lixoft SAS, a Simulations Plus company). One thousand replicates of those simulations were performed, for each replicate estimated PBPK model parameters were sampled from the distribution of the estimates, which was multivariate normal with means being the final point estimates and variance–covariance matrix being and the Fisher Information Matrix (i.e., which reflects the uncertainty in estimates).

Blood concentrations were converted to plasma concentrations by dividing them by the blood plasma concentration ratio. Ceftazidime blood to plasma concentration ratio was 0.82 (PK-Sim (43) prediction), while it was 0.57 for avibactam (44). This transformation enabled calculation of plasma PK parameters such as area under the curve (AUC) and clearance (CL)

From those simulations, the AUC from 0 to 100 h in unbound plasma (AUC_unbound plasma_), muscle interstitium (AUC_muscle interstitium_), and cortex interstitium (AUC_cortex interstitium_) was computed by direct integration of the unbound plasma, muscle interstitium, and cortex interstitium concentrations, respectively. The median and 95% CI of AUCs and AUC ratios AUC_muscle interstitium_/AUC_unbound plasma_ and AUC_cortex interstitium_/AUC_unbound plasma_ were computed and reported.

#### Prediction of human concentrations

Physiological parameters of the final animal PBPK model were substituted by human physiological parameters taken from the literature (see supplemental material for values). For kidney permeability parameters that were estimated, the values estimated from pig data were used.

Simulx (Simulx 2024R1, Lixoft SAS, a Simulations Plus company) was used to perform simulations of expected CAZ and AVI unbound concentrations in plasma and kidney interstitium of a single 70 kg healthy volunteer with a creatinine clearance of 120 mL/min after administration of CZA at doses 2 g/0.5 g and 1 g/0.25 g (CAZ/AVI) every 8 h as 2 h IV infusions over 4 days (arbitrary time selected to ensure that steady state was attained). To compare the ability of our PBPK model to predict unbound plasma concentrations in humans, 1,000 simulations of unbound plasma concentrations of 70 kg a healthy volunteer with a creatinine clearance of 120 mL/min were performed with the Li et al. (9) population PK model using the distribution of PK parameters described in their publication.

From the simulations, %fT > MIC based on unbound CAZ concentrations in plasma and kidney interstitium was computed for MICs ranging from 1 to 128 mg/L. %fT >1 mg/L based on unbound AVI concentrations in plasma and kidney interstitium were also computed.

## SUPPLEMENTAL MATERIAL

Supplemental material aac.01518-24-s0001.docx Model building.

ASM does not own the copyrights to Supplemental Material that may be linked to, or accessed through, an article. The authors have granted ASM a non-exclusive, world-wide license to publish the Supplemental Material files. Please contact the corresponding author directly for reuse.
