# Supplementary material for: Prediction of higher ceftazidime–avibactam concentrations in the human renal interstitium compared with unbound plasma using a minimal physiologically based pharmacokinetic model developed in rats and pigs through microdialysis
Source: Antimicrob Agents Chemother. 2025 Feb 6;69(3):e01518-24. doi: 10.1128/aac.01518-24 (PMC11881572; doi:10.1128/aac.01518-24)
Supplement: Supplemental material — Model building. [file aac.01518-24-s0001.docx]

**Supplemental materials**

# Model building

Detailed model equations can be found in the Monolix model files attached. All parameters used in the models are also provided in spreadsheet format.

## General structure

The body was split into 8 compartments Figure S1:

- Blood was split into two compartments, venous and arterial blood
- Lungs, adipose tissue and liver were represented as homogenous compartments to which drug distribution would be perfusion limited (*i.e.* it was assumed that any molecule that reaches the compartment instaneously distributes to the whole organ)
- Muscles were split into 3 subcompartments representing vascular, interstitial and cellular spaces. Thus, drug distribution was permeability limited. Its structure will be further described below.
- Kidneys were split into 4 anatomical compartments which were further split into subcompartments. Its structure will be further described below.

| 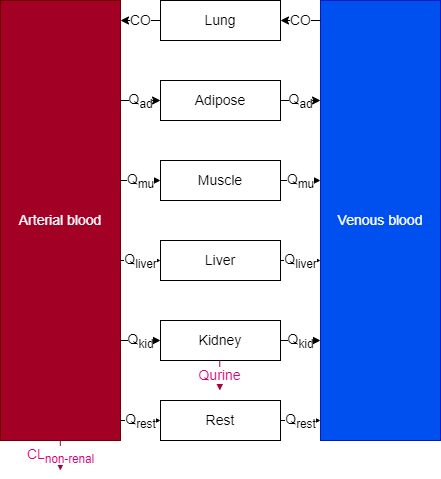  Figure S1: General structure of the PBPK model. CO: cardiac output, Q_ad_: blood flow to adipose tissue, Q_mu_: blood flow to muscles, Q_liver_: blood flow to liver, Q_kid_: blood flow to kidneys, Q_rest_: blood flow to the rest of body compartment, Q_urine_: urinary flow, CL_non-renal_: non renal clearance. |
| --- |

### Blood, lungs, adipose and liver compartments

For each compartment volumes and blood flows were fixed to values found in the literature see [Table S1](#tbl-rat-physio-param), [Table S2](#tbl-pig-physio-param) and [Table S3](#tbl-human-physio-param) below.

| Table S1: Rat physiological parameters used in the PBPK model. TBW: Total body weight CO: Cardiac output   \| Description \| Value \| Unit \| Reference \| \| --- \| --- \| --- \| --- \| \| Cardiac blood output \| 1.78 × 10^1^ \| L/h/kg TBW \| ([1](#Xa6b07a901129dc87654eeaae7608981755c312e)) \| \| Relative blood volume \| 5.40 × 10^−2^ \| L/kg TBW \| ([1](#Xa6b07a901129dc87654eeaae7608981755c312e)) \| \| Relative adipose blood flow \| 5.41 × 10^−3^ \| fraction of CO \| ([1](#Xa6b07a901129dc87654eeaae7608981755c312e)) \| \| Relative adipose volume \| 4.00 × 10^−2^ \| L/kg TBW \| ([1](#Xa6b07a901129dc87654eeaae7608981755c312e)) \| \| Relative liver blood flow \| 1.86 × 10^−1^ \| fraction of CO \| ([1](#Xa6b07a901129dc87654eeaae7608981755c312e)) \| \| Relative liver volume \| 7.84 × 10^−2^ \| L/kg TBW \| ([1](#Xa6b07a901129dc87654eeaae7608981755c312e)) \| \| Relative lung volume \| 8.40 × 10^−3^ \| L/kg TBW \| ([1](#Xa6b07a901129dc87654eeaae7608981755c312e)) \| \| Arterial blood volume relative to total blood volume \| 3.03 × 10^−1^ \| fraction of total blood volume \| ([2](#Xd8c820fb01da94f42cc2d09bf35390f1229bda7)) \| \| Venous blood volume relative to total blood volume \| 6.97 × 10^−1^ \| fraction of total blood volume \| ([2](#Xd8c820fb01da94f42cc2d09bf35390f1229bda7)) \| |
| --- | --- | --- | --- | --- | --- | --- | --- | --- | --- | --- | --- | --- | --- | --- | --- | --- | --- | --- | --- | --- | --- | --- | --- | --- | --- | --- | --- | --- | --- | --- | --- | --- | --- | --- | --- | --- | --- | --- | --- | --- |

| Table S2: Pig physiological parameters used in the PBPK model. TBW: Total body weight CO: Cardiac output   \| Description \| Value \| Unit \| Reference \| \| --- \| --- \| --- \| --- \| \| Relative adipose volume \| 1.54 × 10^−1^ \| L/kg TBW \| ([3](#ref-linPhysiologicalParameterValues2020a)) \| \| Relative blood volume \| 4.12 × 10^−2^ \| L/kg TBW \| ([3](#ref-linPhysiologicalParameterValues2020a)) \| \| Relative liver volume \| 2.04 × 10^−2^ \| L/kg TBW \| ([3](#ref-linPhysiologicalParameterValues2020a)) \| \| Relative lung volume \| 9.00 × 10^−3^ \| L/kg TBW \| ([3](#ref-linPhysiologicalParameterValues2020a)) \| \| Relative liver blood flow \| 2.43 × 10^−1^ \| fraction of CO \| ([3](#ref-linPhysiologicalParameterValues2020a)) \| \| Relative adipose blood flow \| 1.10 × 10^−1^ \| fraction of CO \| ([4](#X054438cc5f7b7f2d7e672c04b21925f9abe3d09)) \| \| Arterial blood volume relative to total blood volume \| 3.30 × 10^−1^ \| fraction of total blood volume \| ([5](#ref-vielPopulationWBPBPKModel2018a)) \| \| Venous blood volume relative to total blood volume \| 6.70 × 10^−1^ \| fraction of total blood volume \| ([5](#ref-vielPopulationWBPBPKModel2018a)) \| \| Cardiac output \| 8.70 \| L/h/kg TBW \| ([3](#ref-linPhysiologicalParameterValues2020a)) \| |
| --- | --- | --- | --- | --- | --- | --- | --- | --- | --- | --- | --- | --- | --- | --- | --- | --- | --- | --- | --- | --- | --- | --- | --- | --- | --- | --- | --- | --- | --- | --- | --- | --- | --- | --- | --- | --- | --- | --- | --- | --- |

| Table S3: Human physiological parameters used in the PBPK model. TBW: Total body weight CO: Cardiac output   \| Description \| Value \| Unit \| Reference \| \| --- \| --- \| --- \| --- \| \| Relative adipose volume \| 1.43 × 10^−1^ \| L/kg TBW \| ([1](#Xa6b07a901129dc87654eeaae7608981755c312e)) \| \| Relative blood volume \| 7.43 × 10^−2^ \| L/kg TBW \| ([1](#Xa6b07a901129dc87654eeaae7608981755c312e)) \| \| Relative liver volume \| 2.41 × 10^−2^ \| L/kg TBW \| ([1](#Xa6b07a901129dc87654eeaae7608981755c312e)) \| \| Relative lung volume \| 1.67 × 10^−2^ \| L/kg TBW \| ([1](#Xa6b07a901129dc87654eeaae7608981755c312e)) \| \| Relative liver blood flow \| 2.59 × 10^−1^ \| fraction of CO \| ([1](#Xa6b07a901129dc87654eeaae7608981755c312e)) \| \| Relative adipose blood flow \| 4.64 × 10^−2^ \| fraction of CO \| ([1](#Xa6b07a901129dc87654eeaae7608981755c312e)) \| \| Arterial blood volume relative to total blood volume \| 3.30 × 10^−1^ \| fraction of total blood volume \| ([5](#ref-vielPopulationWBPBPKModel2018a)) \| \| Venous blood volume relative to total blood volume \| 6.70 × 10^−1^ \| fraction of total blood volume \| ([5](#ref-vielPopulationWBPBPKModel2018a)) \| \| Cardiac output \| 4.80 \| L/h/kg TBW \| ([1](#Xa6b07a901129dc87654eeaae7608981755c312e)) \| |
| --- | --- | --- | --- | --- | --- | --- | --- | --- | --- | --- | --- | --- | --- | --- | --- | --- | --- | --- | --- | --- | --- | --- | --- | --- | --- | --- | --- | --- | --- | --- | --- | --- | --- | --- | --- | --- | --- | --- | --- | --- |

Ceftazidime and avibactam physicochemical parameters ([Table S4](#tbl-cfz-chem-param) and [Table S6](#tbl-avi-chem-param) for ceftazidime and avibactam respetcively) found in literature were used to compute partition coefficients with the Rodgers and Rowland method ([6](#Xbf06ba932260574af5aaba042b2a44cd00132ab)). Partition coefficients can be found in [Table S5](#tbl-cfz-kp-param) and [Table S7](#tbl-avi-kp-param) for ceftazidime and avibactam respectively.

| Table S4: Ceftazidime physico-chemical parameters used in the PBPK model   \| Description \| Value \| Unit \| Reference \| \| --- \| --- \| --- \| --- \| \| Molecular weight \| 5.47 × 10^2^ \| g/mol \| ([7](#Xb830cd9751918209270dc99ffd46ba6a7747eef)) \| \| log10 octanol:water partition coefficient \| −2.65 \| Unitless \| ([7](#Xb830cd9751918209270dc99ffd46ba6a7747eef)) \| \| pKa of the first acid group \| 2.40 \| Unitless \| ([7](#Xb830cd9751918209270dc99ffd46ba6a7747eef)) \| \| pKa of the second acid group \| 4.26 \| Unitless \| ([7](#Xb830cd9751918209270dc99ffd46ba6a7747eef)) \| \| Plasma unbound fraction \| 9.00 × 10^−1^ \| Unitless \| ([7](#Xb830cd9751918209270dc99ffd46ba6a7747eef)) \| \| Blood/plasma ratio \| 8.20 × 10^−1^ \| Unitless \| ([2](#Xd8c820fb01da94f42cc2d09bf35390f1229bda7)) \| |
| --- | --- | --- | --- | --- | --- | --- | --- | --- | --- | --- | --- | --- | --- | --- | --- | --- | --- | --- | --- | --- | --- | --- | --- | --- | --- | --- | --- | --- |

| Table S5: Ceftazidime partition coefficient parameters used in the PBPK model   \| Description \| Value \| Unit \| Reference \| \| --- \| --- \| --- \| --- \| \| Adipose partition coefficient \| 1.28 × 10^−1^ \| Unitless \| Computed \| \| Liver partition coefficient \| 2.01 × 10^−1^ \| Unitless \| Computed \| \| Lung partition coefficient \| 3.48 × 10^−1^ \| Unitless \| Computed \| \| Muscle partition coefficient \| 1.68 × 10^−1^ \| Unitless \| Computed \| \| Muscle interstitial to plasma partition coefficient \| 9.00 × 10^−1^ \| Unitless \| Computed \| \| Muscle cell to interstitial partition coefficient \| 7.77 × 10^−2^ \| Unitless \| Computed \| |
| --- | --- | --- | --- | --- | --- | --- | --- | --- | --- | --- | --- | --- | --- | --- | --- | --- | --- | --- | --- | --- | --- | --- | --- | --- | --- | --- | --- | --- |

| Table S6: Avibactam physico-chemical parameters used in the PBPK model   \| Description \| Value \| Unit \| Reference \| \| --- \| --- \| --- \| --- \| \| Molecular weight \| 2.65 × 10^2^ \| g/mol \| Pubchem - https://pubchem.ncbi.nlm.nih.gov/compound/Avibactam \| \| log10 octanol:water partition coefficient \| −3.60 \| Unitless \| ([8](#X915d801dc6ed012f981ccd6677fd04c22672b6a)) \| \| pKa of the first acid group \| 0.00 \| Unitless \| ([8](#X915d801dc6ed012f981ccd6677fd04c22672b6a)) \| \| Plasma unbound fraction \| 9.20 × 10^−1^ \| Unitless \| ([8](#X915d801dc6ed012f981ccd6677fd04c22672b6a)) \| \| Blood/plasma ratio \| 5.50 × 10^−1^ \| Unitless \| ([8](#X915d801dc6ed012f981ccd6677fd04c22672b6a)) \| |
| --- | --- | --- | --- | --- | --- | --- | --- | --- | --- | --- | --- | --- | --- | --- | --- | --- | --- | --- | --- | --- | --- | --- | --- | --- |

| Table S7: Avibactam partition coefficient parameters used in the PBPK model   \| Description \| Value \| Unit \| Reference \| \| --- \| --- \| --- \| --- \| \| Adipose partition coefficient \| 1.33 × 10^−1^ \| Unitless \| Computed \| \| Liver partition coefficient \| 3.19 × 10^−1^ \| Unitless \| Computed \| \| Lung partition coefficient \| 4.43 × 10^−1^ \| Unitless \| Computed \| \| Muscle partition coefficient \| 2.96 × 10^−1^ \| Unitless \| Computed \| \| Muscle interstitial to plasma partition coefficient \| 9.20 × 10^−1^ \| Unitless \| Computed \| \| Muscle cell to interstitial partition coefficient \| 2.31 × 10^−1^ \| Unitless \| Computed \| |
| --- | --- | --- | --- | --- | --- | --- | --- | --- | --- | --- | --- | --- | --- | --- | --- | --- | --- | --- | --- | --- | --- | --- | --- | --- | --- | --- | --- | --- |

### Muscle compartment

The muscle compartment was split into three subcompartments representing the vascular, interstitial and cellular spaces with passive transfer between all three spaces (Figure S2). For each subcompartment volumes and blood flows were fixed to values found in the literature see [Table S8](#tbl-rat-mu-param), [Table S9](#tbl-pig-mu-param) and [Table S10](#tbl-human-mu-param) for rat, pig and human respectively.

| 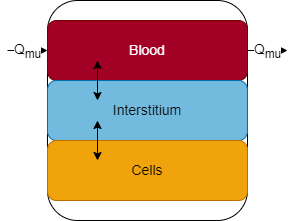  Figure S2: Detailed structure of the muscle compartment. Q_mu_: blood flow to muscles. |
| --- |

| Table S8: Rat muscle physiological parameters used in the PBPK model. TBW: Total body weight CO: Cardiac output   \| Description \| Value \| Unit \| Reference \| \| --- \| --- \| --- \| --- \| \| Relative muscle blood flow \| 1.01 × 10^−1^ \| fraction of CO \| ([1](#Xa6b07a901129dc87654eeaae7608981755c312e)) \| \| Relative muscle volume \| 5.80 × 10^−1^ \| L/kg TBW \| ([1](#Xa6b07a901129dc87654eeaae7608981755c312e)) \| \| Relative muscle vasculature volume \| 2.60 × 10^−2^ \| fraction of muscle volume \| ([9](#Xc6b1e44ef46f71ed7ea0220ee7f1f5c6f9d7358)) \| \| Relative muscle interstitial volume \| 1.20 × 10^−1^ \| fraction of muscle volume \| ([9](#Xc6b1e44ef46f71ed7ea0220ee7f1f5c6f9d7358)) \| \| Relative muscle cellular volume \| 8.54 × 10^−1^ \| fraction of muscle volume \| ([9](#Xc6b1e44ef46f71ed7ea0220ee7f1f5c6f9d7358)) \| |
| --- | --- | --- | --- | --- | --- | --- | --- | --- | --- | --- | --- | --- | --- | --- | --- | --- | --- | --- | --- | --- | --- | --- | --- | --- |

| Table S9: Pig muscle physiological parameters used in the PBPK model. TBW: Total body weight CO: Cardiac output   \| Description \| Value \| Unit \| Reference \| \| --- \| --- \| --- \| --- \| \| Relative muscle volume \| 3.63 × 10^−1^ \| L/kg TBW \| ([3](#ref-linPhysiologicalParameterValues2020a)) \| \| Relative muscle blood flow \| 3.42 × 10^−1^ \| fraction of CO \| ([3](#ref-linPhysiologicalParameterValues2020a)) \| \| Relative muscle vasculature volume \| 1.50 × 10^−2^ \| fraction of muscle volume \| ([3](#ref-linPhysiologicalParameterValues2020a)) \| \| Relative muscle cellular volume \| 8.25 × 10^−1^ \| fraction of muscle volume \| ([10](#ref-tanComparativeProteomicAnalysis2023a)) \| \| Relative muscle interstitial volume \| 1.60 × 10^−1^ \| fraction of muscle volume \| ([10](#ref-tanComparativeProteomicAnalysis2023a)) \| |
| --- | --- | --- | --- | --- | --- | --- | --- | --- | --- | --- | --- | --- | --- | --- | --- | --- | --- | --- | --- | --- | --- | --- | --- | --- |

| Table S10: Human muscle physiological parameters used in the PBPK model. TBW: Total body weight CO: Cardiac output   \| Description \| Value \| Unit \| Reference \| \| --- \| --- \| --- \| --- \| \| Relative muscle volume \| 5.00 × 10^−1^ \| L/kg TBW \| ([1](#Xa6b07a901129dc87654eeaae7608981755c312e)) \| \| Relative muscle blood flow \| 1.34 × 10^−1^ \| fraction of CO \| ([1](#Xa6b07a901129dc87654eeaae7608981755c312e)) \| \| Relative muscle vasculature volume \| 3.00 × 10^−2^ \| fraction of muscle volume \| ([2](#Xd8c820fb01da94f42cc2d09bf35390f1229bda7)) \| \| Relative muscle cellular volume \| 8.20 × 10^−1^ \| fraction of muscle volume \| ([2](#Xd8c820fb01da94f42cc2d09bf35390f1229bda7)) \| \| Relative muscle interstitial volume \| 1.60 × 10^−1^ \| fraction of muscle volume \| ([2](#Xd8c820fb01da94f42cc2d09bf35390f1229bda7)) \| |
| --- | --- | --- | --- | --- | --- | --- | --- | --- | --- | --- | --- | --- | --- | --- | --- | --- | --- | --- | --- | --- | --- | --- | --- | --- |

Diffusion between vascular and interstitial subcompartments was assumed to be instantaneous. Diffusion between interstitial and cellular subcompartment was characterized by an apparent permeability parameter which was estimated using PK-Sim 11 ([2](#Xd8c820fb01da94f42cc2d09bf35390f1229bda7))

| Table S11: Ceftazidime permeability parameters used in the PBPK model   \| Description \| Value \| Unit \| Reference \| \| --- \| --- \| --- \| --- \| \| Apparent permeability from plasma to muscle interstitial space \| 3.60 × 10^4^ \| dm/h \| Assumed instantaneous diffusion \| \| Apparent permeability from muscle interstitial space to muscle intracellular space \| 1.45 × 10^−8^ \| dm/h \| ([2](#Xd8c820fb01da94f42cc2d09bf35390f1229bda7)) \| \| Apparent permeability across LLC-PK1 cells in the apical to basal direction \| 5.18 × 10^−4^ \| dm/h \| Experimental mesurement \| \| Apparent permeability across LLC-PK1 cells in the basal to apical direction \| 1.12 × 10^−3^ \| dm/h \| Experimental mesurement \| \| Apparent permeability from plasma to kidney interstitial space \| 3.60 × 10^4^ \| dm/h \| Assumed instantaneous diffusion \| |
| --- | --- | --- | --- | --- | --- | --- | --- | --- | --- | --- | --- | --- | --- | --- | --- | --- | --- | --- | --- | --- | --- | --- | --- | --- |

| Table S12: Avibactam permeability parameters used in the PBPK model   \| Description \| Value \| Unit \| Reference \| \| --- \| --- \| --- \| --- \| \| Apparent permeability from plasma to muscle interstitial space \| 3.60 × 10^4^ \| dm/h \| Assumed instantaneous diffusion \| \| Apparent permeability from muscle interstitial space to muscle intracellular space \| 1.25 × 10^−7^ \| dm/h \| ([2](#Xd8c820fb01da94f42cc2d09bf35390f1229bda7)) \| \| Apparent permeability across LLC-PK1 cells in the apical to basal direction \| 9.11 × 10^−4^ \| dm/h \| Experimental mesurement \| \| Apparent permeability across LLC-PK1 cells in the basal to apical direction \| 1.88 × 10^−3^ \| dm/h \| Experimental mesurement \| \| Apparent permeability from plasma to kidney interstitial space \| 3.60 × 10^4^ \| dm/h \| Assumed instantaneous diffusion \| |
| --- | --- | --- | --- | --- | --- | --- | --- | --- | --- | --- | --- | --- | --- | --- | --- | --- | --- | --- | --- | --- | --- | --- | --- | --- |

### Kidney compartment

The kidney was split into 4 anatomical compartments, the cortex, the outer medulla and two inner medulla compartments Figure S3.

Each anatomical compartment was split into subcompartments representing tubules, interstitial space and blood vessels.

The proximal tubule was split into 3 subcompartments.

The collecting duct was split into 9 subcompartments, one compartment for each nephron fusion event.

Transcellular permeability across tubular cells was fixed to the *in vitro* apparent permeability values measured on LLC-PK1 cells.

| 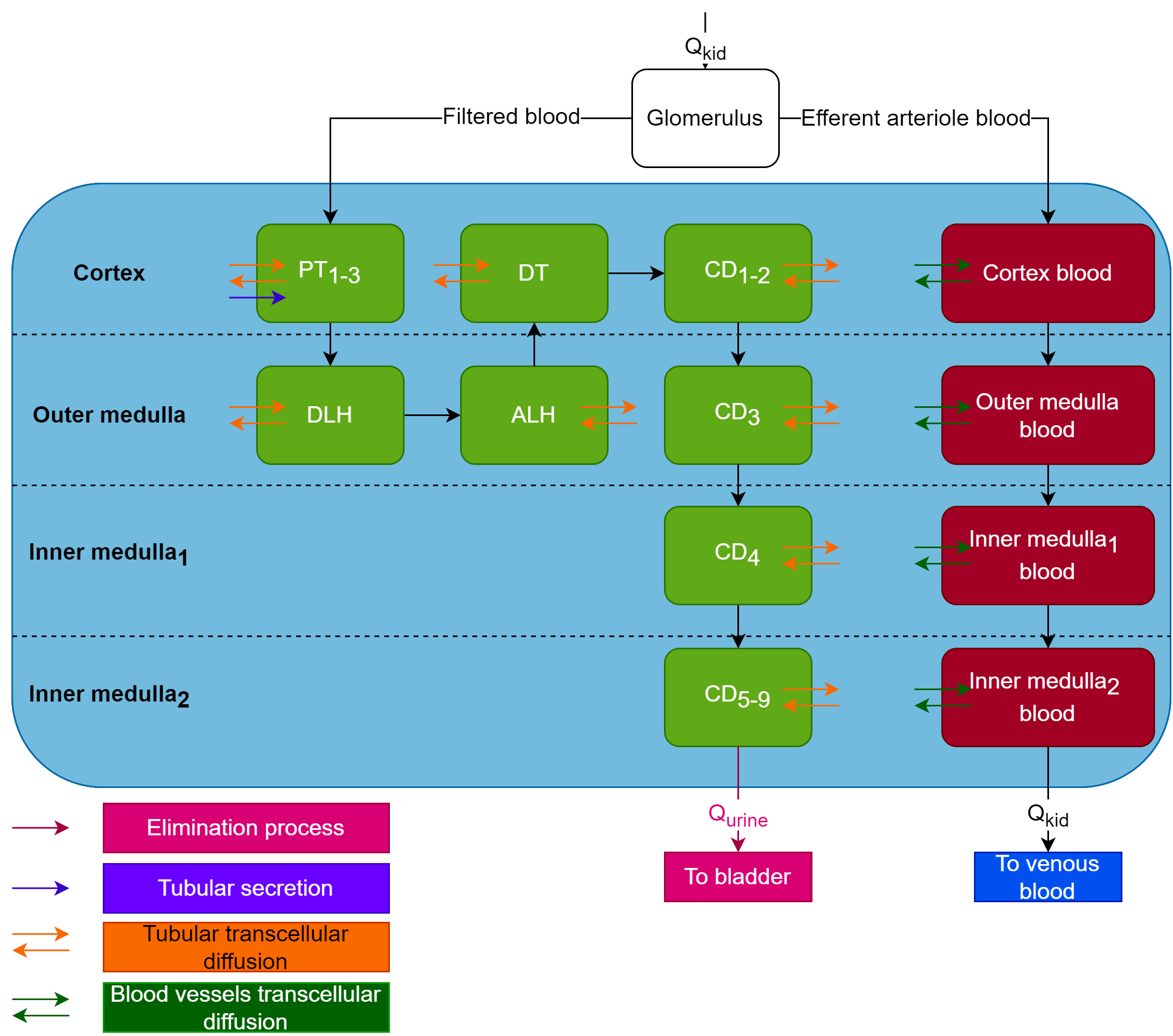  Figure S3: Detailed structure of the kidney compartment. Q_kid_: blood flow to kidneys, Qurine: urinary flow, PT: proximal tubule, DLH: descending loop of Henlé, ALH: ascending loop of Henlé, DT: distal tubule, CD: collecting duct. Green: tubular lumen compartments, light blue: interstitium compartments, red: blood vessels compartments. |
| --- |

#### Tubular compartments

Urine flow into the first lumen subcompartment of the proximal tubule was set to the glomerular filtration rate. Urine flows into each subsequent lumen subcompartment were reduced by the reabsorpbtion of water:

$$Q_{urine,i}=Q_{urine,i-1}\times\left( 1-f_{reabsorb} \right)$$

Where :

- i is the number of the tubule lumen subcompartment
- Q_urine,i_ is the urine flow into lumen subcompartment i

Lumen subcompartments radii were taken from the litterature when available. Collecting duct lumen subcompartments 2 to 9 radii were not found in litterature. They were computed using the formula derived by Scotcher *et al.*. The reader is referred to their supplemental material (especially equation S1.1) for a detailed explanation ([11](#X27a5ab85562cfcf2fc4916a0dc7968cb9617d10)).

It was assumed that lumen subcompartment radii were proportional to the cubic root of weight (which is equivalent to the common assumption that volumes are proportional to bodyweight) while lumen subcompartment lengths were assumed to be constant. A proportionality constant was computed to adjust lumen subcompartment radii to modifications in body weight in order to compute subcompartment volumes:

$$r_{lumen,i,j}=cst_{lumen,rad,WT}\times WT_{j}^{1/3}$$

$$cst_{lumen,rad,WT}=\frac{r_{lumen,i,ref}}{WT_{ref}^{1/3}}$$

Where :

- i is the number of the tubule lumen subcompartment
- j is an individual animal identifier
- r_lumen,i,j_ is the radius of lumen subcompartment i for animal j
- cst_lumen,rad,WT_ is the constant to adjust radius to body weight
- WT_j_ is the weight of animal j
- r_lumen,i,ref_ is the reference radius of lumen subcompartment i
- WT_ref_ is the reference weight of animals from which r_lumen,i,ref_ has been defined

Volumes of tubular lumen subcompartments were computed from lumen subcompartment radius and length with the assumption that lumen subcompartments were circular right cylinders:

$$V_{lumen,i,j}=\pi\times r_{lumen,i,j}^{2}\times L_{lumen,i,j}$$

Where :

- i is the number of the tubule lumen subcompartment
- j is an individual animal identifier
- V_lumen,i,j_ is the volume of lumen subcompartment i for animal j
- r_lumen,i,j_ is the radius of lumen subcompartment i for animal j
- L_lumen,,ji_ is the length of lumen subcompartment i for animal j

Surface areas of tubular lumen subcompartments were computed under the assumtion that the lumen subcompartments were circular right cylinders :

$$SA_{lumen,i,j}=2\times\pi\times r_{lumen,i,j}\times L_{lumen,i,j}$$

Where :

- SA_lumen,i,j_ is the surface area of tubular cell lumen subcompartment i for animal j
- r_lumen,i,j_ is the radius of lumen subcompartment i for animal j
- L_lumen,,ji_ is the length of lumen subcompartment i for animal j

#### Lumen cell volumes

Although lumen cells are not present as compartments in our model, computing their volume was necessary to then compute interstitial and blood volumes. It was assumed that cell thickness was 20% of lumen radius. Cellular volumes were computed under the assumption that tubules were circular right cylinders as explained before.

#### Blood compartments

Blood flows in kidney blood compartments was assumed to be constant and fixed to the kidney blood flow.

The total volume of blood and interstitial compartments was computed as the total kidney volume minus total kidney lumen and total kidney cellular volume. Blood compartments were assumed to occupy 3/4 of the non-lumen, non-cellular space :

$$V_{ki,blood,j}=\frac{3}{4}\times\left( V_{ki,j}-\left( V_{ki,lumen,j}+V_{ki,cell,j} \right) \right)$$

Where:

- j is an individual animal identifier
- V_ki,blood,j_ is total volume of kidney blood compartments for animal j
- V_ki,j_ is total volume of kidney for animal j
- V_ki,lumen,j_ is total volume of kidney tubular lumen compartments for animal j
- V_ki,cell,j_ is total volume of kidney tubular cell compartments for animal j

For a given blood subcompartment, its volume was assumed to be proportional to the volume of the corresponding lumen subcompartment:

$$V_{blood,i,j}=V_{ki,blood,j}\times\frac{V_{lumen,i,j}}{V_{ki,j}}$$

Where:

- j is an individual animal identifier
- V_ki,blood,j_ is total volume of kidney blood compartments for animal j
- V_ki,lumen,i,j_ is volume of kidney tubular lumen subcompartment i for animal j
- V_ki,j_ is total volume of kidney for animal j

For a given blood subcompartment, its length was assumed to be equal to the length of the corresponding lumen subcompartment. From its volume and its length, its radius was computed assuming that it was a right straight cylinder:

$$r_{blood,i,j}=\sqrt{\frac{V_{blood,i,j}}{\pi\times L_{lumen,i,j}}}$$

Where :

- i is the number of the blood subcompartment
- j is an individual animal identifier
- r_blood,i,j_ is the radius of blood subcompartment i for animal j
- L_lumen,i,j_ is the length of blood subcompartment i for animal j

Surface areas of blood subcompartments were computed under the assumtion that the blood subcompartments were circular right cylinders :

$$SA_{blood,i,j}=2\times\pi\times r_{blood,i,j}\times L_{lumen,i,j}$$

Where :

- SA_blood,i,j_ is the surface area of tubular cell blood subcompartment i for animal j
- r_blood,i,j_ is the radius of blood subcompartment i for animal j
- L_lumen,i,j_ is the length of blood subcompartment i for animal j

#### Interstitial compartments

The total volume of blood and interstitial compartments was computed as the total kidney volume minus total kindey lumen and total kidney cellular volume. Interstitial compartments were assumed to occupy 1/4 of the non-lumen, non-cellular space :

$$V_{ki,interst,j}=\frac{1}{4}\times\left( V_{ki,j}-\left( V_{ki,lumen,j}+V_{ki,cell,j} \right) \right)$$

Where:

- j is an individual animal identifier
- V_ki,interst,j_ is total volume of kidney interstitial compartments for animal j
- V_ki,j_ is total volume of kidney for animal j
- V_ki,lumen,j_ is total volume of kidney tubular lumen compartments for animal j
- V_ki,cell,j_ is total volume of kidney tubular cell compartments for animal j

For a given interstitial subcompartment, its volume was assumed to be proportional to the volume of the corresponding lumen subcompartment:

$$V_{interst,i,j}=V_{ki,interst,j}\times\frac{V_{lumen,i,j}}{V_{ki,j}}$$

Where:

- j is an individual animal identifier
- V_ki,interst,i,j_ is volume of kidney interstitial compartment i for animal j
- V_ki,interst,j_ is total volume of kidney interstitial compartments for animal j
- V_ki,lumen,i,j_ is volume of kidney tubular lumen subcompartment i for animal j
- V_ki,j_ is total volume of kidney for animal j.

Interstitial subcompartments were grouped into 4 anatomical subcompartments, cortex, outer medulla, inner medulla 1 and inner medulla 2. Their volumes were calculated as follows:

Cortex interstitial volume:

$$V_{interst,cortex,j}=V_{interst,PT1,j}+V_{interst,PT2,j}+V_{interst,PT3,j}+V_{interst,DT,j}+V_{interst,CD1,j}+V_{interst,CD2,j}$$

Where :

- j is an individual animal identifier
- V_interst,cortex,j_ is the cortex interstitial subcompartment volume for animal j
- V_interst,PT1,j_ is the proximal tubule interstitial subcompartment 1 volume for animal j
- V_interst,PT2,j_ is the proximal tubule interstitial subcompartment 2 volume for animal j
- V_interst,PT3,j_ is the proximal tubule interstitial subcompartment 3 volume for animal j
- V_interst,DT,j_ is the distal tubule interstitial subcompartment volume for animal j
- V_interst,CD1,j_ is the cortex collecting duct interstitial subcompartment volume for animal j
- V_interst,CD2,j_ is the collecting duct interstitial subcompartment 2 volume for animal j

Outer medulla interstitial volume:

$$V_{interst,outerMed,j}=V_{interst,LH1,j}+V_{interst,LH2,j}+V_{interst,CD3,j}$$

Where :

- j is an individual animal identifier
- V_interst,outerMed,j_ is the outer medulla interstitial subcompartment volume for animal j
- V_interst,LH1,j_ is the descending loop of Henlé interstitial subcompartment volume for animal j
- V_interst,LH2,j_ is the ascending loop of Henlé interstitial subcompartment volume for animal j
- V_interst,CD3,j_ is the collecting duct interstitial subcompartment 3 volume for animal j

Inner medulla 1 interstitial volume:

$$V_{interst,innerMed1,j}=V_{interst,CD4,j}$$

Where :

- j is an individual animal identifier
- V_interst,innerMed1,j_ is the inner medulla 1 interstitial subcompartment volume for animal j
- V_interst,CD4,j_ is the collecting duct interstitial subcompartment 4 volume for animal j

Inner medulla 2 interstitial volume:

$$V_{interst,innerMed2,j}=V_{interst,CD5,j}+V_{interst,CD6,j}+V_{interst,CD7,j}+V_{interst,CD8,j}+V_{interst,CD9,j}$$

Where :

- j is an individual animal identifier
- V_interst,innerMed2,j_ is the inner medulla 2 interstitial subcompartment volume for animal j
- V_interst,CD5,j_ is the collecting duct interstitial subcompartment 5 volume for animal j
- V_interst,CD6,j_ is the collecting duct interstitial subcompartment 6 volume for animal j
- V_interst,CD7,j_ is the collecting duct interstitial subcompartment 7 volume for animal j
- V_interst,CD8,j_ is the collecting duct interstitial subcompartment 8 volume for animal j
- V_interst,CD9,j_ is the collecting duct interstitial subcompartment 9 volume for animal j

#### Table of kidney parameters

| Table S13: Rat kidney physiological parameters used in the PBPK model. TBW: Total body weight CO: Cardiac output   \| Description \| Value \| Unit \| Reference \| \| --- \| --- \| --- \| --- \| \| General \| \| \| \| \| GFR per weight \| 4.74 × 10^−1^ \| L/h/kg TBW \| ([12](#ref-carraraSimplifiedMethodMeasure2016a)) \| \| Relative kidney blood flow \| 1.24 × 10^−1^ \| fraction of CO \| ([1](#Xa6b07a901129dc87654eeaae7608981755c312e)) \| \| Relative kidney volume \| 1.48 × 10^−2^ \| L/kg TBW \| ([1](#Xa6b07a901129dc87654eeaae7608981755c312e)) \| \| Relative kidney weight \| 8.00 × 10^−3^ \| fraction of TBW \| ([1](#Xa6b07a901129dc87654eeaae7608981755c312e)) \| \| Urine formation rate \| 1.68 × 10^−3^ \| L/h/kg TBW \| ([13](#ref-liPhysiologicallyBasedSilico2020a)) \| \| Proximal tubule \| \| \| \| \| Proximal tubule fraction of water reabsorbed in section \| 7.00 × 10^−1^ \| Unitless \| ([13](#ref-liPhysiologicallyBasedSilico2020a)) \| \| Proximal tubule length \| 1.38 × 10^1^ \| mm \| ([14](#ref-lettsNephronMorphometryMice2017a)) \| \| Proximal tubule lumen (inner) radius \| 1.46 × 10^−2^ \| mm \| ([14](#ref-lettsNephronMorphometryMice2017a)) \| \| Proximal tubule number per kidney \| 2.90 × 10^4^ \| Unitless \| ([14](#ref-lettsNephronMorphometryMice2017a)) \| \| Loop of Henlé \| \| \| \| \| Ascending Loop of Henlé fraction of water reabsorbed in section \| 0.00 \| Unitless \| ([13](#ref-liPhysiologicallyBasedSilico2020a)) \| \| Ascending Loop of Henlé length \| 7.47 \| mm \| ([14](#ref-lettsNephronMorphometryMice2017a)) \| \| Ascending Loop of Henlé lumen (inner) radius \| 6.60 × 10^−3^ \| mm \| ([14](#ref-lettsNephronMorphometryMice2017a)) \| \| Ascending Loop of Henlé number per kidney \| 2.90 × 10^4^ \| Unitless \| ([14](#ref-lettsNephronMorphometryMice2017a)) \| \| Descending Loop of Henlé fraction of water reabsorbed in section \| 1.00 × 10^−1^ \| Unitless \| ([13](#ref-liPhysiologicallyBasedSilico2020a)) \| \| Descending Loop of Henlé length \| 2.69 \| mm \| ([14](#ref-lettsNephronMorphometryMice2017a)) \| \| Descending Loop of Henlé lumen (inner) radius \| 7.90 × 10^−3^ \| mm \| ([14](#ref-lettsNephronMorphometryMice2017a)) \| \| Descending Loop of Henlé number per kidney \| 2.90 × 10^4^ \| Unitless \| ([14](#ref-lettsNephronMorphometryMice2017a)) \| \| Distal tubule \| \| \| \| \| Distal Tubule fraction of water reabsorbed in section \| 1.00 × 10^−1^ \| Unitless \| ([13](#ref-liPhysiologicallyBasedSilico2020a)) \| \| Distal Tubule length \| 3.54 \| mm \| ([14](#ref-lettsNephronMorphometryMice2017a)) \| \| Distal Tubule lumen (inner) radius \| 1.29 × 10^−2^ \| mm \| ([14](#ref-lettsNephronMorphometryMice2017a)) \| \| Distal Tubule number per kidney \| 2.90 × 10^4^ \| Unitless \| ([14](#ref-lettsNephronMorphometryMice2017a)) \| \| Collecting Duct \| \| \| \| \| Collecting Duct cortex fraction of water reabsorbed in section \| 6.00 × 10^−2^ \| Unitless \| ([13](#ref-liPhysiologicallyBasedSilico2020a)) \| \| Collecting Duct cortex length \| 1.00 \| mm \| ([15](#X9ac7b0ca546b859b6768ac4303ad810a70461af)) \| \| Collecting Duct cortex lumen (inner) radius \| 1.00 × 10^−2^ \| mm \| ([15](#X9ac7b0ca546b859b6768ac4303ad810a70461af)) \| \| Collecting Duct medulla fraction of water reabsorbed in section \| 3.00 × 10^−2^ \| Unitless \| ([13](#ref-liPhysiologicallyBasedSilico2020a)) \| \| Collecting Duct medulla length \| 7.50 \| mm \| ([13](#ref-liPhysiologicallyBasedSilico2020a)) \| |
| --- | --- | --- | --- | --- | --- | --- | --- | --- | --- | --- | --- | --- | --- | --- | --- | --- | --- | --- | --- | --- | --- | --- | --- | --- | --- | --- | --- | --- | --- | --- | --- | --- | --- | --- | --- | --- | --- | --- | --- | --- | --- | --- | --- | --- | --- | --- | --- | --- | --- | --- | --- | --- | --- | --- | --- | --- | --- | --- | --- | --- | --- | --- | --- | --- | --- | --- | --- | --- | --- | --- | --- | --- | --- | --- | --- | --- | --- | --- | --- | --- | --- | --- | --- | --- | --- | --- | --- | --- | --- | --- | --- | --- | --- | --- | --- | --- | --- | --- | --- | --- | --- | --- | --- | --- | --- | --- | --- | --- | --- | --- | --- | --- | --- | --- | --- | --- | --- | --- | --- | --- | --- | --- | --- | --- | --- | --- | --- | --- |

| Table S14: Pig kidney physiological parameters used in the PBPK model. TBW: Total body weight CO: Cardiac output   \| Description \| Value \| Unit \| Reference \| \| --- \| --- \| --- \| --- \| \| General \| \| \| \| \| GFR per weight \| 1.38 × 10^−1^ \| L/h/kg TBW \| ([16](#ref-lodrupAssociationRenalFunction2008)) \| \| Relative kidney blood flow \| 1.14 × 10^−1^ \| fraction of CO \| ([3](#ref-linPhysiologicalParameterValues2020a)) \| \| Relative kidney volume \| 3.70 × 10^−3^ \| L/kg TBW \| ([3](#ref-linPhysiologicalParameterValues2020a)) \| \| Urine formation rate per weight \| 4.07 × 10^−3^ \| L/h/kg TBW \| ([16](#ref-lodrupAssociationRenalFunction2008)) \| \| Proximal tubule \| \| \| \| \| Proximal tubule fraction of water reabsorbed in section \| 7.00 × 10^−1^ \| Unitless \| ([13](#ref-liPhysiologicallyBasedSilico2020a)) \| \| Proximal tubule length \| 1.80 × 10^1^ \| mm \| ([13](#ref-liPhysiologicallyBasedSilico2020a)) \| \| Proximal tubule lumen (inner) radius \| 2.06 × 10^−2^ \| mm \| ([17](#ref-sikarwarMICROMETRYKIDNEYLARGE2016)) \| \| Proximal tubule number per kidney \| 9.03 × 10^5^ \| Unitless \| ([18](#ref-mollerDimensionalChangesProximal1987)) \| \| Loop of Henlé \| \| \| \| \| Ascending Loop of Henlé fraction of water reabsorbed in section \| 0.00 \| Unitless \| ([13](#ref-liPhysiologicallyBasedSilico2020a)) \| \| Ascending Loop of Henlé length \| 7.47 \| mm \| ([13](#ref-liPhysiologicallyBasedSilico2020a)) \| \| Ascending Loop of Henlé lumen (inner) radius \| 9.77 × 10^−3^ \| mm \| ([17](#ref-sikarwarMICROMETRYKIDNEYLARGE2016)) \| \| Ascending Loop of Henlé number per kidney \| 9.03 × 10^5^ \| Unitless \| ([18](#ref-mollerDimensionalChangesProximal1987)) \| \| Descending Loop of Henlé fraction of water reabsorbed in section \| 1.00 × 10^−1^ \| Unitless \| ([13](#ref-liPhysiologicallyBasedSilico2020a)) \| \| Descending Loop of Henlé length \| 5.37 \| mm \| ([13](#ref-liPhysiologicallyBasedSilico2020a)) \| \| Descending Loop of Henlé lumen (inner) radius \| 1.35 × 10^−2^ \| mm \| ([17](#ref-sikarwarMICROMETRYKIDNEYLARGE2016)) \| \| Descending Loop of Henlé number per kidney \| 9.03 × 10^5^ \| Unitless \| ([18](#ref-mollerDimensionalChangesProximal1987)) \| \| Distal tubule \| \| \| \| \| Distal Tubule fraction of water reabsorbed in section \| 1.00 × 10^−1^ \| Unitless \| ([13](#ref-liPhysiologicallyBasedSilico2020a)) \| \| Distal Tubule length \| 3.54 \| mm \| ([13](#ref-liPhysiologicallyBasedSilico2020a)) \| \| Distal Tubule lumen (inner) radius \| 1.62 × 10^−2^ \| mm \| ([17](#ref-sikarwarMICROMETRYKIDNEYLARGE2016)) \| \| Distal Tubule number per kidney \| 9.03 × 10^5^ \| Unitless \| ([18](#ref-mollerDimensionalChangesProximal1987)) \| \| Collecting duct \| \| \| \| \| Collecting Duct cortex fraction of water reabsorbed in section \| 6.00 × 10^−2^ \| Unitless \| ([13](#ref-liPhysiologicallyBasedSilico2020a)) \| \| Collecting Duct cortex length \| 2.00 \| mm \| ([13](#ref-liPhysiologicallyBasedSilico2020a)) \| \| Collecting Duct cortex lumen (inner) radius \| 1.80 × 10^−2^ \| mm \| ([17](#ref-sikarwarMICROMETRYKIDNEYLARGE2016)) \| \| Collecting Duct medulla fraction of water reabsorbed in section \| 3.00 × 10^−2^ \| Unitless \| ([13](#ref-liPhysiologicallyBasedSilico2020a)) \| \| Collecting Duct medulla length \| 1.50 × 10^1^ \| mm \| ([13](#ref-liPhysiologicallyBasedSilico2020a)) \| |
| --- | --- | --- | --- | --- | --- | --- | --- | --- | --- | --- | --- | --- | --- | --- | --- | --- | --- | --- | --- | --- | --- | --- | --- | --- | --- | --- | --- | --- | --- | --- | --- | --- | --- | --- | --- | --- | --- | --- | --- | --- | --- | --- | --- | --- | --- | --- | --- | --- | --- | --- | --- | --- | --- | --- | --- | --- | --- | --- | --- | --- | --- | --- | --- | --- | --- | --- | --- | --- | --- | --- | --- | --- | --- | --- | --- | --- | --- | --- | --- | --- | --- | --- | --- | --- | --- | --- | --- | --- | --- | --- | --- | --- | --- | --- | --- | --- | --- | --- | --- | --- | --- | --- | --- | --- | --- | --- | --- | --- | --- | --- | --- | --- | --- | --- | --- | --- | --- | --- | --- | --- | --- | --- | --- | --- |

| Table S15: Human kidney physiological parameters used in the PBPK model. TBW: Total body weight CO: Cardiac output   \| Description \| Value \| Unit \| Reference \| \| --- \| --- \| --- \| --- \| \| General \| \| \| \| \| GFR per weight \| 1.07 × 10^−1^ \| L/h/kg TBW \| ([13](#ref-liPhysiologicallyBasedSilico2020a)) \| \| Relative kidney blood flow \| 2.21 × 10^−1^ \| fraction of CO \| ([1](#Xa6b07a901129dc87654eeaae7608981755c312e)) \| \| Relative kidney volume \| 4.00 × 10^−3^ \| L/kg TBW \| ([1](#Xa6b07a901129dc87654eeaae7608981755c312e)) \| \| Urine formation rate per weight \| 1.07 × 10^−3^ \| L/h/kg TBW \| ([13](#ref-liPhysiologicallyBasedSilico2020a)) \| \| Proximal tubule \| \| \| \| \| Proximal tubule fraction of water reabsorbed in section \| 7.00 × 10^−1^ \| Unitless \| ([13](#ref-liPhysiologicallyBasedSilico2020a)) \| \| Proximal tubule length \| 1.80 × 10^1^ \| mm \| ([13](#ref-liPhysiologicallyBasedSilico2020a)) \| \| Proximal tubule lumen (inner) radius \| 3.00 × 10^−2^ \| mm \| ([13](#ref-liPhysiologicallyBasedSilico2020a)) \| \| Proximal tubule number per kidney \| 1.00 × 10^6^ \| Unitless \| ([13](#ref-liPhysiologicallyBasedSilico2020a)) \| \| Loop of Henlé \| \| \| \| \| Ascending Loop of Henlé fraction of water reabsorbed in section \| 0.00 \| Unitless \| ([13](#ref-liPhysiologicallyBasedSilico2020a)) \| \| Ascending Loop of Henlé length \| 7.00 \| mm \| ([13](#ref-liPhysiologicallyBasedSilico2020a)) \| \| Ascending Loop of Henlé lumen (inner) radius \| 1.25 × 10^−2^ \| mm \| ([13](#ref-liPhysiologicallyBasedSilico2020a)) \| \| Ascending Loop of Henlé number per kidney \| 1.00 × 10^6^ \| Unitless \| ([13](#ref-liPhysiologicallyBasedSilico2020a)) \| \| Descending Loop of Henlé fraction of water reabsorbed in section \| 1.00 × 10^−1^ \| Unitless \| ([13](#ref-liPhysiologicallyBasedSilico2020a)) \| \| Descending Loop of Henlé length \| 7.00 \| mm \| ([13](#ref-liPhysiologicallyBasedSilico2020a)) \| \| Descending Loop of Henlé lumen (inner) radius \| 1.10 × 10^−2^ \| mm \| ([13](#ref-liPhysiologicallyBasedSilico2020a)) \| \| Descending Loop of Henlé number per kidney \| 1.00 × 10^6^ \| Unitless \| ([13](#ref-liPhysiologicallyBasedSilico2020a)) \| \| Distal tubule \| \| \| \| \| Distal Tubule fraction of water reabsorbed in section \| 1.00 × 10^−1^ \| Unitless \| ([13](#ref-liPhysiologicallyBasedSilico2020a)) \| \| Distal Tubule length \| 5.50 \| mm \| ([13](#ref-liPhysiologicallyBasedSilico2020a)) \| \| Distal Tubule lumen (inner) radius \| 1.50 × 10^−2^ \| mm \| ([13](#ref-liPhysiologicallyBasedSilico2020a)) \| \| Distal Tubule number per kidney \| 1.00 × 10^6^ \| Unitless \| ([13](#ref-liPhysiologicallyBasedSilico2020a)) \| \| Collecting duct \| \| \| \| \| Collecting Duct cortex fraction of water reabsorbed in section \| 6.00 × 10^−2^ \| Unitless \| ([13](#ref-liPhysiologicallyBasedSilico2020a)) \| \| Collecting Duct cortex length \| 8.00 \| mm \| ([13](#ref-liPhysiologicallyBasedSilico2020a)) \| \| Collecting Duct cortex lumen (inner) radius \| 2.50 × 10^−2^ \| mm \| ([13](#ref-liPhysiologicallyBasedSilico2020a)) \| \| Collecting Duct medulla fraction of water reabsorbed in section \| 3.00 × 10^−2^ \| Unitless \| ([13](#ref-liPhysiologicallyBasedSilico2020a)) \| \| Collecting Duct medulla length \| 1.40 × 10^1^ \| mm \| ([13](#ref-liPhysiologicallyBasedSilico2020a)) \| |
| --- | --- | --- | --- | --- | --- | --- | --- | --- | --- | --- | --- | --- | --- | --- | --- | --- | --- | --- | --- | --- | --- | --- | --- | --- | --- | --- | --- | --- | --- | --- | --- | --- | --- | --- | --- | --- | --- | --- | --- | --- | --- | --- | --- | --- | --- | --- | --- | --- | --- | --- | --- | --- | --- | --- | --- | --- | --- | --- | --- | --- | --- | --- | --- | --- | --- | --- | --- | --- | --- | --- | --- | --- | --- | --- | --- | --- | --- | --- | --- | --- | --- | --- | --- | --- | --- | --- | --- | --- | --- | --- | --- | --- | --- | --- | --- | --- | --- | --- | --- | --- | --- | --- | --- | --- | --- | --- | --- | --- | --- | --- | --- | --- | --- | --- | --- | --- | --- | --- | --- | --- | --- | --- | --- | --- |

##

## Bibliography

1. Davies B, Morris T. 1993. [Physiological Parameters in Laboratory Animals and Humans](https://doi.org/10.1023/A:1018943613122). Pharmaceutical Research 10:1093–1095.

2. Willmann S, Lippert J, Sevestre M, Solodenko J, Fois F, Schmitt W. 2003. [PK-Sim®: a physiologically based pharmacokinetic ‘whole-body’ model](https://doi.org/10.1016/S1478-5382(03)02342-4). BIOSILICO 1:121–124.

3. Lin Z, Li M, Wang Y-S, Tell LA, Baynes RE, Davis JL, Vickroy TW, Riviere JE. 2020. [Physiological parameter values for physiologically based pharmacokinetic models in food-producing animals. Part I: Cattle and swine](https://doi.org/10.1111/jvp.12861). Journal of Veterinary Pharmacology and Therapeutics 43:385–420.

4. Lautz LS, Dorne JLCM, Oldenkamp R, Hendriks AJ, Ragas AMJ. 2020. [Generic physiologically based kinetic modelling for farm animals: Part I. Data collection of physiological parameters in swine, cattle and sheep](https://doi.org/10.1016/j.toxlet.2019.10.021). Toxicology Letters 319:95–101.

5. Viel A, Henri J, Bouchène S, Laroche J, Rolland J-G, Manceau J, Laurentie M, Couet W, Grégoire N. 2018. [A Population WB-PBPK Model of Colistin and its Prodrug CMS in Pigs: Focus on the Renal Distribution and Excretion](https://doi.org/10.1007/s11095-018-2379-4). Pharmaceutical Research 35:92.

6. Rodgers T, Rowland M. 2006. [Physiologically based pharmacokinetic modelling 2: predicting the tissue distribution of acids, very weak bases, neutrals and zwitterions](https://doi.org/10.1002/jps.20502). Journal of Pharmaceutical Sciences 95:1238–1257.

7. Cui C, Li X, Liang H, Hou Z, Tu S, Dong Z, Yao X, Zhang M, Zhang X, Li H, Zuo X, Liu D. 2021. [Physiologically based pharmacokinetic model of renally cleared antibacterial drugs in Chinese renal impairment patients](https://doi.org/10.1002/bdd.2258). Biopharmaceutics & Drug Disposition 42:24–34.

8. Zhou W, Johnson T, Xu H, Cheung S, Bui K, Li J, Al-Huniti N, Zhou D. 2016. [Predictive Performance of Physiologically Based Pharmacokinetic and Population Pharmacokinetic Modeling of Renally Cleared Drugs in Children](https://doi.org/10.1002/psp4.12101). CPT: Pharmacometrics & Systems Pharmacology 5:475–483.

9. Kawai R, Lemaire M, Steimer J-L, Bruelisauer A, Niederberger W, Rowland M. 1994. [Physiologically based pharmacokinetic study on a cyclosporin derivative, SDZ IMM 125](https://doi.org/10.1007/BF02353860). Journal of Pharmacokinetics and Biopharmaceutics 22:327–365.

10. Tan X, He Y, He Y, Yan Z, Chen J, Zhao R, Sui X, Zhang L, Du X, Irwin DM, Zhang S, Li B. 2023. [Comparative Proteomic Analysis of Glycolytic and Oxidative Muscle in Pigs](https://doi.org/10.3390/genes14020361). Genes 14:361.

11. Scotcher D, Jones C, Rostami-Hodjegan A, Galetin A. 2016. [Novel minimal physiologically-based model for the prediction of passive tubular reabsorption and renal excretion clearance](https://doi.org/10.1016/j.ejps.2016.03.018). European Journal of Pharmaceutical Sciences: Official Journal of the European Federation for Pharmaceutical Sciences 94:59–71.

12. Carrara F, Azzollini N, Nattino G, Corna D, Villa S, Cerullo D, Zoja C, Abrante B, Luis-Lima S, Porrini E, Cannata A, Ferrari S, Fois M, Stucchi N, Gaspari F. 2016. [Simplified Method to Measure Glomerular Filtration Rate by Iohexol Plasma Clearance in Conscious Rats](https://doi.org/10.1159/000445843). Nephron 133:62–70.

13. Li Z, Litchfield J, Tess DA, Carlo AA, Eng H, Keefer C, Maurer TS. 2020. [A Physiologically Based in Silico Tool to Assess the Risk of Drug-Related Crystalluria](https://doi.org/10.1021/acs.jmedchem.9b01995). Journal of Medicinal Chemistry 63:6489–6498.

14. Letts RFR, Zhai X-Y, Bhikha C, Grann BL, Blom NB, Thomsen JS, Rubin DM, Christensen EI, Andreasen A. 2017. [Nephron morphometry in mice and rats using tomographic microscopy](https://doi.org/10.1152/ajprenal.00207.2016). American Journal of Physiology-Renal Physiology 312:F210–F229.

15. Niederalt C, Wendl T, Kuepfer L, Claassen K, Loosen R, Willmann S, Lippert J, Schultze-Mosgau M, Winkler J, Burghaus R, Bräutigam M, Pietsch H, Lengsfeld P. 2013. [Development of a Physiologically Based Computational Kidney Model to Describe the Renal Excretion of Hydrophilic Agents in Rats](https://doi.org/10.3389/fphys.2012.00494). Frontiers in Physiology 3.

16. Lødrup AB, Karstoft K, Dissing TH, Nyengaard JR, Pedersen M. 2008. [The association between renal function and structural parameters: A pig study](https://doi.org/10.1186/1471-2369-9-18). BMC Nephrology 9:18.

17. Sikarwar S, Mathur R, Joshi S, Beniwal G. 2016. MICROMETRY IN THE KIDNEY OF LARGE WHITE YORKSHIRE PIG (SUS SCROFA) UNDER LIGHT MICROSCOPE. Veterinary Practitioner 17.

18. Møller JC. 1987. [Dimensional changes of proximal tubules and cortical capillaries in chronic obstructive renal disease: A light microscopic morphometric analysis](https://doi.org/10.1007/BF00713520). Virchows Archiv A Pathological Anatomy and Histopathology 410:153–158.
